# Nucleosome positioning and histone modifications define relationships between regulatory elements and nearby gene expression in breast epithelial cells

**DOI:** 10.1186/1471-2164-15-331

**Published:** 2014-05-02

**Authors:** Suhn Kyong Rhie, Dennis J Hazelett, Simon G Coetzee, Chunli Yan, Houtan Noushmehr, Gerhard A Coetzee

**Affiliations:** Department of Preventive Medicine, Keck School of Medicine, University of Southern California, Los Angeles, CA 90089 USA; Norris Cancer Center, Keck School of Medicine, University of Southern California, Los Angeles, CA 90089 USA; Department of Urology, Keck School of Medicine, University of Southern California, Los Angeles, CA 90089 USA; Department of Genetics, Faculty of Medicine in Ribeirão Preto, University of São Paulo, 14049-900 São Paulo, Brazil; Urology, Microbiology and Preventive Medicine, USC/Norris Cancer Center, NOR 6411, Keck School of Medicine, 1441 Eastlake Ave, Los Angeles, CA 90089-9176 USA

**Keywords:** Breast epithelial cells, Epigenetics, Nucleosomes, Enhancers, Promoters, Transcription factors, Genes

## Abstract

**Background:**

The precise nature of how cell type specific chromatin structures at enhancer sites affect gene expression is largely unknown. Here we identified cell type specific enhancers coupled with gene expression in two different types of breast epithelial cells, HMEC (normal breast epithelial cells) and MDAMB231 (triple negative breast cancer cell line).

**Results:**

Enhancers were defined by modified neighboring histones [using chromatin immunoprecipitation followed by sequencing (ChIP-seq)] and nucleosome depletion [using formaldehyde-assisted isolation of regulatory elements followed by sequencing (FAIRE-seq)]. Histone modifications at enhancers were related to the expression levels of nearby genes up to 750 kb away. These expression levels were correlated with enhancer status (poised or active), defined by surrounding histone marks. Furthermore, about fifty percent of poised and active enhancers contained nucleosome-depleted regions. We also identified response element motifs enriched at these enhancer sites that revealed key transcription factors (e.g. TP63) likely involved in regulating breast epithelial enhancer-mediated gene expression. By utilizing expression data, potential target genes of more than 600 active enhancers were identified. These genes were involved in proteolysis, epidermis development, cell adhesion, mitosis, cell cycle, and DNA replication.

**Conclusions:**

These findings facilitate the understanding of epigenetic regulation specifically, such as the relationships between regulatory elements and gene expression and generally, how breast epithelial cellular phenotypes are determined by cell type specific enhancers.

**Electronic supplementary material:**

The online version of this article (doi:10.1186/1471-2164-15-331) contains supplementary material, which is available to authorized users.

## Background

The human genome has 22 chromosome pairs and 2 sex chromosomes. This single genome gives rise to several hundred distinct cell types including epithelial, neuronal, lymphocytic, germ, etc. In 2003, the human genome was completely sequenced, and it ushered in a new understanding of the breadth and diversity of primary coding sequences [1]. However, the specific mechanism(s), explaining how one genome gives rise to a large number of diverse cells and tissues, remains unclear. What has become apparent is that dynamic gene expression levels determined by regulatory networks, whose building blocks are in turn governed by epigenetic chromatin states, may explain how cellular phenotypes are defined.

Genomic DNA is organized into chromatin; 147 base pairs of DNA are wrapped around two copies of each of the core histone proteins, namely H2A, H2B, H3, and H4, forming a single octameric nucleosome core particle. Chromatin is found in two forms: hetero- and euchromatin. Heterochromatin is highly condensed, forming tightly packaged chromatin. In this compact state, a high density of repetitive DNA elements such as satellite DNA and transposable elements are found, and the dense packing of DNA makes it less likely for transcription factors to bind [2]. In euchromatin, by contrast, chromatin is relatively ‘open’ or loosely packaged, forming ‘beads on a string’ structures. In this open form, gene regulatory proteins may easily bind to the DNA, and nuclear processes such as transcription can be performed and influenced by dynamic nucleosome positioning [3]. Nucleosome position and histone marks play important roles to demarcate regulatory elements such as promoters, enhancers, and repressed regions.

Enhancers are non-directional regulatory elements that control gene expression at a distance on linear DNA [4]; i.e. they reside in so-called non-coding DNA regions such as intergenic regions and introns. Several histone marks (H3K4me1, H3K4me2, H3K9Ac, H3K14Ac, H3K27Ac) [5, 6] and nucleosome-depleted regions have been shown to correlate or to be associated with regions that display enhancer activity; nucleosome-depleted regions can be visualized by DNaseI-sensitivity and/or Formaldehyde-Assisted Isolation of Regulatory Elements (FAIRE) [7]. Enhancers generally contain DNA-recognition motifs for transcription factors, which regulate gene expression upon binding, and loop to the transcriptional start sites of target genes [4].

Recently, a number of studies investigated the roles of regulatory elements, distal from transcription start sites (TSS) (i.e. enhancers), and revealed that the multiplicity of cell states was determined by tissue-specific distal regulatory elements [7, 8]. In the present study, we hypothesized that cell type specific enhancers may distinguish breast epithelial cells with different phenotypes. In order to identify such specific enhancers related to the maintenance of distinct breast epithelial phenotypes, human mammary epithelial cell (HMEC) and breast cancer cell line (MDAMB231) were chosen to study, representing extremes of breast epithelial cells. In addition, both cell types were selected because they have been shown to be estrogen receptor (ER) negative: MDAMB231 is a triple-negative breast cancer cell line [9–12]. It is known that breast cancer (BCa) can arise from the transformation of a normal ER-negative mammary epithelial cell [13]. Previous studies support the notion that epithelial to messenchymal transition (EMT) in breast cancer is linked to the triple negative (ER-, PR-, HER2-) breast cancer subgroup and even to cancer stem cells [14–16]. However, it has been shown that ER signaling can regulate EMT [17]. The presence of ER in certain BCa cells may complicate analyses by having differently regulated genes and changes in chromatin structures upon ER binding to regulatory elements [18, 19]. By using ChIP-seq and FAIRE-seq methods, we identified enhancers, which were unique to each cell type of breast epithelial cells (i.e. HMEC and MDAMB231) -- we specifically excluded ER + cell lines from our analyses for reasons alluded to above. The expression profiles of genes in these two types of cells along with histone marks were investigated in order to identify the target genes of cell type specific enhancers. In our view, information gained using the approaches described in this study will aid the understanding of epigenetic regulation and breast cancer biology.

## Results and discussion

### Characterization of breast epithelial cell type specific enhancers

**Figure 1:**
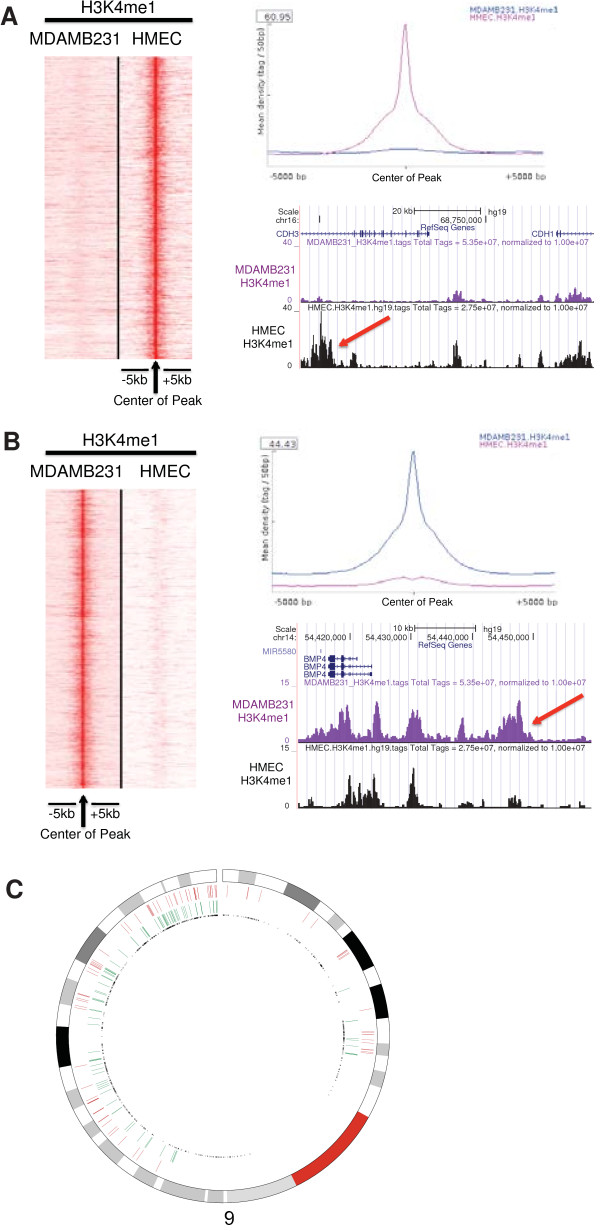
**Cell type specific enhancer loci identification in breast epithelial cells (HMEC and MDAMB231). (A)** H3K4me1 ChIP-seq tags from both cells at the center of HMEC specific enhancer loci (HSEL) were graphed in the heatmap (red: higher density) (left). Mean density of H3K4me1 ChIP-seq tags from both cells at the HSEL (top right). An example of the HSEL was located in the intron of *CDH3* gene, near *CDH1* gene (red arrow) (bottom right). **(B)** H3K4me1 ChIP-seq tags from both cells at the center of MDAMB231 specific enhancer loci (MSEL) were graphed in the heatmap (red: higher density) (left). Mean density of H3K4me1 ChIP-seq tags from both cells at the MSEL (top right). An example of the MSEL located 20 kb upstream of the *BMP4* gene (red arrow) (bottom right). **(C)** Genomic distribution of cell type specific enhancer loci in chromosome 9 (red: HSEL, green: MSEL, black: transcription start sites).

Identification of enhancer specific histone modification mark, H3K4me1 by ChIP-seq, revealed 110,715 peaks in both cell types. Unlike other histone marks such as H3K4me3 and H3K27Ac, the overlap of H3K4me1 peaks between two cell types were only 22 percent for HMEC and 70 percent for MDAMB231 (Additional file 1: Figure S1 and Additional file 2: Table S1, Table S2, Table S3). This indicated that large numbers of enhancers defined by this histone modification mark were unique to either cell type, and there were relatively more H3K4me1 marked enhancers in HMEC than MDAMB231. For further downstream analysis, we selected the top 2,000 most robust cell type specific H3K4me1 sites (Additional file 2: Table S4, Table S5) ranked by fold-change of ChIP-seq tags between two cell types using findPeaks software from HOMER ( http://homer.salk.edu/homer/) (see Methods) [20]. In order to verify the method used to identify cell type specific enhancers, we compared our HMEC specific enhancer loci (HSEL) with enhancers classified by using a multivariate Hidden Markov Model in HMEC from ENCODE database [21]. Ninety-seven percent of HSEL (n = 1940) overlapped with HMM enhancers (42 percent with weak enhancers in HMM, 58 percent with strong enhancers in HMM), indicating that our method is applicable. At HMEC specific enhancer loci (HSEL), H3K4me1 marks were highly enriched in HMEC, compared to MDAMB231 with more than 60 fold higher mean density of H3K4me1 ChIP-seq tags (Figure 1A). To illustrate, we selected a distinct HSEL located in the intron of *CDH3* and 60 kb upstream of the *CDH1*’s transcription start site. *CDH1* and *CDH3* encode calcium-dependent cell-cell adhesion glycoproteins, E-cadherin and P-cadherin, respectively. E-cadherin is expressed in most normal epithelial cells, and loss of E-cadherin expression has been observed in various tumors including breast cancer [22–24]. Unlike E-cadherin, P-cadherin is expressed in limited epithelial tissues, and it has been reported that this gene is aberrantly expressed in breast cancer cells [25–28]. Similarly, we identified MDAMB231 specific enhancer loci (MSEL) unique in the MDAMB231 cell type (Figure 1B). An example of such a MSEL site is located 20 kb upstream from the transcription start site of *BMP4*, which encodes a bone morphogenetic protein 4 (BMP4). BMPs are first detected in extracts of bone and they regulate various developmental processes [29]. BMP4 is involved in development of mammary gland and required for migration and invasion of breast cancer [29–31].

Next, we investigated the genomic distributions of the HSEL and MSEL. Most of the HSEL and MSEL specific marks were found in introns and intergenic regions (Additional file 1: Figure S2A, S3A). The HSEL and MSEL were distributed evenly throughout the genome, with no specific chromosomal enrichment (Additional file 1: Figure S2B and Figure S3B). Remarkably, although the two sets of putative enhancer groups were mutually exclusive (by selection), some of them still appear to cluster in the same genomic regions as exemplified by chromosome 9 (Figure 1C). These data seem to be consistent with the finding that cell type specific enhancers with related functions cluster in chromosomal regions with correlated gene expressions in different cell types [32]. These speculative enhancer clusters may be related to the high density of TSSs, high density of pioneer protein binding, and/or chromatin structures at these regions. In order to test these possibilities, further investigations near the enhancer clusters such as calculating gene density, transcription factor binding, analyzing DNA methylation, and nuclear lamina associated domains are required.

### Nearby gene expression change by cell type specific enhancers

The next biological question was to determine which genes might be targets of the cell type specific enhancer loci identified above (HSEL and MSEL). To this end, the differentially expressed genes in HMEC and MDAMB231 were analyzed using published data [33] and then matched to the cell-type specific enhancers identified here. Several approaches were used to examine the relationships between cell type specific enhancers and gene expression.

**Figure 2:**
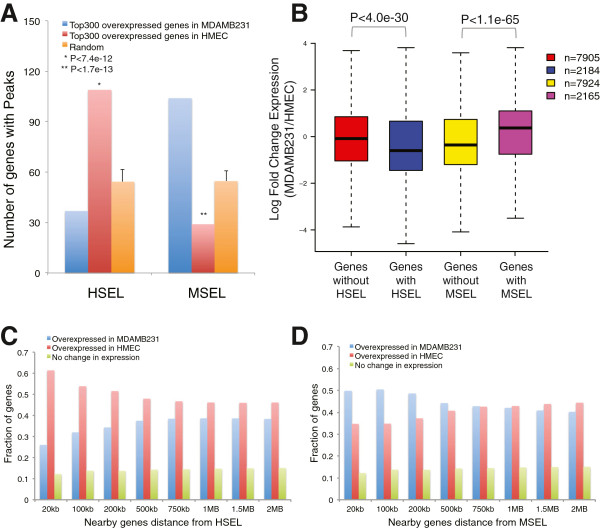
**Expression level of nearby genes of the HSEL and MSEL. (A)** Number of genes with cell type specific enhancer loci (i.e. HSEL, MSEL) from top 300 overexpressed genes in MDAMB231 (blue), top 300 overexpressed genes in HMEC (red), and three randomly selected 300 gene datasets as controls (orange). The enrichment of genes with cell type specific enhancer loci in each group was calculated by performing chi-square test between groups. **(B)** Log fold change of nearby gene expression boxplot for the HSEL and MSEL (in windows of 100 kb). Student t-test was applied between groups in order to calculate p-values. Nearby genes of cell type specific enhancer loci (HSEL **(C)**, MSEL **(D)**) were categorized to three groups; overexpressed genes in MDAMB231 (blue), overexpressed genes in HMEC (red), and no change in gene expression (green), and fraction of genes for each category was graphed. Eight different window sizes were used for the nearby gene distance from cell type specific enhancer loci: from 20 kb windows of cell type specific enhancer loci (±20 kb of HSEL/MSEL) up to 2 Mb windows (±2 Mb of HSEL/MSEL).

In the first approach, we selected the highest confidence cell type specific genes and examined their distribution relative to cell type specific enhancers. For this purpose, genes with a p-value < 0.05 (3,174 genes for HMEC, 2,670 genes for MDAMB231) were ranked based on their fold-change in two cell type. The top 300 differentially expressed genes, which represent about the top 10 percent of the genes, were selected and statistical enrichment analyses were performed, using a fixed-distance metric of 100 kb to the nearest enhancer site. There were twice as many overexpressed genes in HMEC than randomly sampled genes (see Methods), whose transcription start sites were located within 100 kb of HSEL (109 genes out of top 300, which showed higher expression in HMEC than MDAMB231 vs. 54 out of 300 random control genes). Similarly, there were 1.9 times as many overexpressed genes in MDAMB231 than randomly sampled genes, whose transcription start sites were located within 100 kb of MSEL (104 out of top 300 overexpressed genes in MDAMB231 vs. 55 out of 300 random control genes) (Figure 2A). Under the hypothesis of independent assignment of the feature, cell type specific enhancers and overexpression, we found that the number of differentially expressed genes having HSEL would be between 45 and 70 genes (95% confidence interval) by direct simulation (k = 100,000). Similarly, the number of differentially expressed genes having MSEL would be between 42 and 67 (95% confidence interval) if the relationship between expression and cell type specific enhancer was independent. The above reported numbers of 109 and 104 for overexpressed genes (in HMEC and MDAMB231, respectively) with cell type specific enhancers (HSEL and MSEL, respectively) fell outside of these intervals. Conversely, the reported numbers of 37 and 29 for underexpressed genes (i.e. overexpressed genes in the other cell type) in HMEC and MDAMB231, respectively with cell type specific enhancers (HSEL and MSEL, respectively) were below 45 and 42 (95% confidence). Therefore, these data suggest that overexpressed genes in one cell type (HMEC or MDAMB231) are enriched in the vicinity of cell type specific enhancers HSEL and MSEL respectively, and this enrichment is accompanied by a concomitant depletion of cell type specific enhancers from the other cell type.

In a second approach, we centered our analysis on the cell type specific enhancers and compared the expression profile of genes within a 100 kb window on either side of the HSEL or MSEL. If it is indeed the case that there is a relationship between the presence of cell type specific enhancers and differentially expressed genes, then we should be able to detect an effect of such enhancers on nearby gene expression. On average, the expression levels of genes with HSEL (1,418 genes, 2,184 probes) were significantly higher in HMEC than MDAMB231 as indicated by the plotted ratio, compared to control genes, which were not near HSEL (Additional file 1: Figure S4A and Figure 2B). Likewise, the average expression levels of genes with MSEL (1,405 genes, 2,165 probes) were higher in MDAMB231 than HMEC as indicated by the plotted ratio, compared to control genes, which were not near MSEL (Additional file 1: Figure S4B and Figure 2B). In addition, we plotted all the genes within a 100 kb window on either side of the HSEL or MSEL in a scatter plot with x-axis for expression levels in HMEC and y-axis for expression levels in MDAMB231. Among genes within a 100 kb window on either side of the HSEL, there were more overexpressed genes in HMEC than in MDAMB231. Conversely, among genes within a 100 kb window on either side of the MSEL, there were more overexpressed genes in MDAMB231 than in HMEC (Additional file 1: Figure S5). Collectively, these results indicated that cell type specific enhancers were well correlated with differentially expressed genes in both cell types.

In order to infer the physical range over which the presence of cell type specific enhancers influences gene expression levels, we examined the fraction of differentially expressed genes as a function of distance from the enhancer. To this end, genes were grouped in increasing window sizes around the cell type specific enhancers from 20 kb up to 2 Mb. All the genes within given window sizes of cell type specific enhancers were then categorized as either overexpressed in HMEC, overexpressed in MDAMB231, or not differently expressed genes.

Genes nearby HMEC specific enhancers were expressed higher in HMEC compared to MDAMB231, but the effect decreased as the distance between HSEL and nearby genes increased in general. For instance, the overexpressed genes in HMEC were 1.7 times more than overexpressed genes in MDAMB231 within a 100 kb window on either side of these HSEL (p = 4.2e-32) (Figure 2C, Additional file 1: Figure S6A and Additional file 2: Table S6). However, the overexpressed genes in HMEC were only 1.2 times more than overexpressed genes in MDAMB231 within a 750 kb window on either side of these HSEL. There were still relatively more overexpressed genes in HMEC even within a 2 Mb window on either side of the HSEL. This maybe due to the fact that average expression value for all genes probed in the microarray was a little bit higher in HMEC as a possible result of having more enhancers in HMEC, compared to MDAMB231 (Additional file 1: Figure S7). Among control genes, which were not near HSEL, the overexpressed genes in HMEC were about 1.2 times more than overexpressed genes in MDAMB231 regardless of the distance (Additional file 1: Figure S8A). Therefore, we can interpret these results that there were significantly more overexpressed genes in HMEC when the distance from HSEL was smaller than 750 kb. The effect decreased if the window size increased from 750 kb to 2 Mb. When the distance between HSEL and nearby genes was bigger than 750 kb, the fraction of overexpressed genes in HMEC was not statistically significant, compared to control genes (p > 0.17) (Additional file 2: Table S6).

Conversely, more genes nearby MDAMB231 specific enhancers were expressed higher in MDAMB231, compared to HMEC when the window size was smaller than 750 kb. The overexpressed genes in MDAMB231 were 1.4 times more than overexpressed genes in HMEC within a 100 kb window on either side of these MSEL (p = 6.76e-17) (Figure 2D, Additional file 1: Figure S6B). The effect of MSEL on increasing gene expression in MDAMB231 vs. HMEC was most significant when the window size between MSEL and nearby gene was 100 kb (Additional file 2: Table S6). However, the effect decreased as the window size increased. Thus, almost the same number of overexpressed genes in MDAMB231 (n = 1,687) and overexpressed genes in HMEC (n = 1,679) was found within a 750 kb window on either side of these MSEL (p = 0.86) (Figure 2D, Additional file 1: Figure S6B). In control genes, which were not near MSEL (from window size 20 kb to 2 Mb), there were more overexpressed genes in HMEC than overexpressed genes in MDAMB231 regardless of the distance (Additional file 1: Figure S8B and Additional file 2: Table S6).

As another control dataset, we selected 2,000 H3K4me1 sites found in both cell types (called shared enhancers) (Additional file 1: Figure S9 and Additional file 2: Table S7), and investigated gene expression levels near the shared enhancers from window size 20 kb to 2 Mb. Unlike genes near cell type specific enhancers, there were approximately the same number of overexpressed genes in HMEC and overexpressed genes in MDAMB231 across the window size from 20 kb to 2 Mb (Additional file 1: Figure S10, Additional file 2: Table S6). For example, there were 630 overexpressed genes in MDAMB231 and 673 overexpressed genes in HMEC within a 100 kb window of shared enhancers (p > 0.11). Within a 2 Mb window of shared enhancers, 2,206 overexpressed genes in MDAMB231 and 2,258 overexpressed genes in HMEC were found.

As Chepelev et al [34] and Sheffield et al [35] suggested, our findings further supported that on average, cell type specific enhancers seemed to regulate genes closer than 100 kb, but the effect decreased as the gene distance increased: The significance of correlation was evident with nearby genes as far 750 kb from cell type specific enhancers (Figure 2B,C,D, Additional file 1: Figure S11). These findings remained consistent when we investigated genes at each distance interval from window size 20 kb to 2 Mb of enhancers (Additional file 1: Figure S12, Additional file 2: Table S8). Overall, cell type specific enhancers were correlated with nearby genes, which were differentially expressed in both cell types.

### Enhancer status and nearby gene expression level change

Recently, several groups categorized enhancers into a number of functional classes by using histone modifications [36, 37]. From these studies, it became apparent that H3K27Ac marks demarcate active enhancers, whereas H3K4me1 marks define both active and poised enhancers. In other words, enrichment of H3K4me1 and H3K27Ac correlated with active and engaged enhancers [38, 39], whereas poised enhancers (H3K4me1 without H3K27Ac marks) correlated with relatively lower expression levels of the target genes [36].

**Figure 3:**
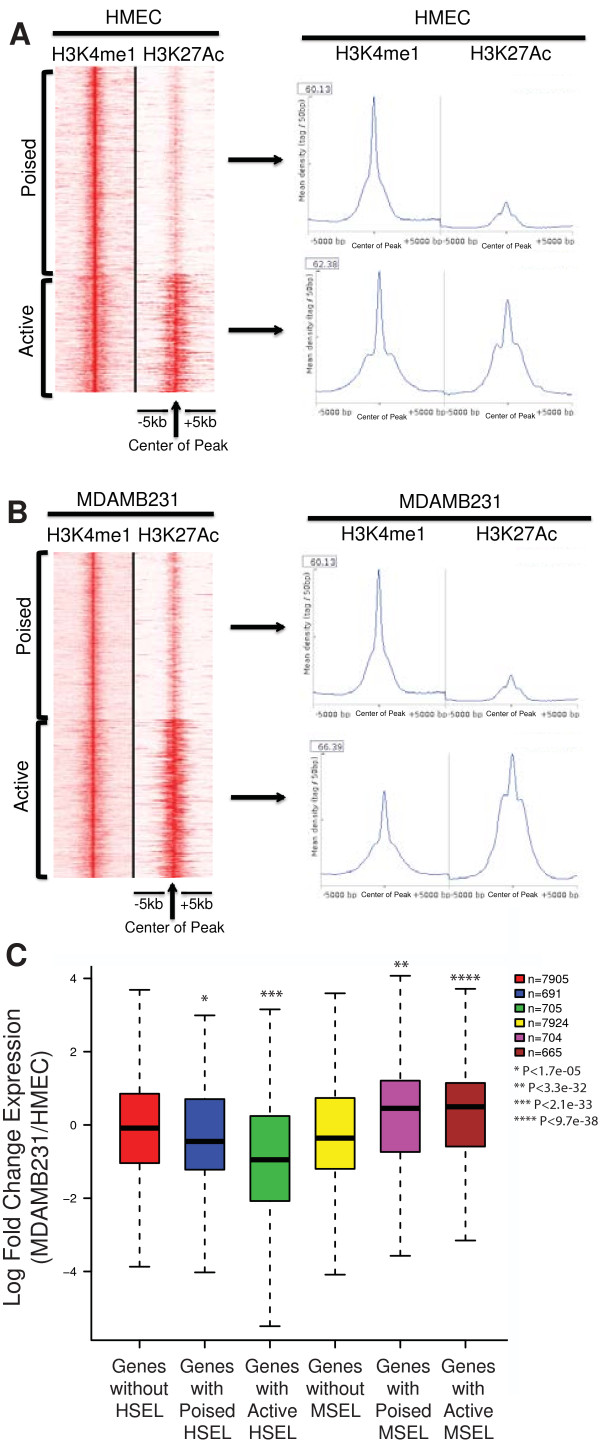
**Poised and active enhancers. (A)** H3K4me1 and H3K27Ac ChIP-seq tags from HMEC at the HSEL were graphed in the heatmap (red: higher density) (left). Mean density of H3K4me1 and H3K27Ac ChIP-seq tags from HMEC at the poised HSEL (top right) and the active HSEL (bottom right) **(B)** H3K4me1 and H3K27Ac ChIP-seq tags from MDAMB231 at the MSEL were graphed in the heatmap (red: higher density) (left). Mean density of H3K4me1 and H3K27Ac ChIP-seq tags from MDAMB231 at the poised MSEL (top right) and the active MSEL (bottom right) **(C)** Log fold change of nearby gene expression boxplot for the poised/active HSEL and poised/active MSEL. Student t-test was applied between groups in order to calculate p-values.

In order to determine whether the expression levels of nearby genes in our study were also correlated with the enhancer status as defined above, we classified HSEL as poised (n = 1,270) or active (n = 730) using K means linear clustering with H3K27Ac ChIP-seq data [40] (Figure 3A, Additional file 2: Table S4). When classifying the MSEL in a similar manner, 1,021 poised MSEL and 979 active MSEL were identified (Figure 3B, Additional file 2: Table S5).

Next, the expression levels of nearby genes (+/-100 kb) of the poised and active enhancer groups as defined above were assessed by randomly selecting 500 poised HSEL, 500 active HSEL, 500 poised MSEL, and 500 active MSEL. On average, genes near poised HSEL were significantly overexpressed in HMEC relative to MDAMB231 (Figure 3C, blue boxplot): genes located outside of HSEL were not differentially expressed (Figure 3C, red boxplot). The average expression levels of genes located near active HSEL were even higher in HMEC than MDAMB231 (Figure 3C, green boxplot). Conversely, on average, genes near poised MSEL were expressed at higher levels in MDAMB231 than in HMEC, and the expression levels in MDAMB231 were even higher for genes near active MSEL (Figure 3C, compare purple and crimson vs. yellow boxplots). Therefore, cell type specific enhancer status defined by histone modifications was positively correlated with cell type specific regulation of nearby gene expression.

### Detection of Nucleosome-depleted regions at both poised and active enhancers

**Figure 4:**
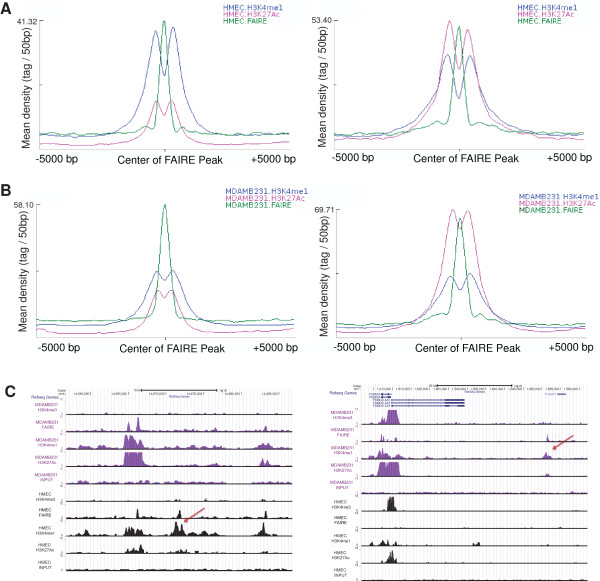
**Poised and active enhancers with FAIRE signals. (A)** Mean density of H3K4me1, H3K27Ac ChIP-seq, and FAIRE-seq tags from HMEC at the center of poised (left) or active (right) HSEL, which were intersected with HMEC FAIRE **(B)** Mean density of H3K4me1, H3K27Ac ChIP-seq, and FAIRE-seq tags from MDAMB231 at the center of poised (left) or active (right) MSEL, which were intersected with MDAMB231 FAIRE **(C)** UCSC genome browser screenshots for examples of poised cell type specific enhancer loci with FAIRE signal (left: poised HSEL, right: poised MSEL)

In order to investigate open chromatin regions at cell-type specific enhancers, we first intersected FAIRE-seq peaks (n = 57,489) from HMEC with the HMEC specific enhancer loci (HSEL). Most of these open chromatin regions in enhancers correspond to nucleosome-depleted regions [7, 41, 42]. Among our 2,000 HSEL, HMEC FAIRE peaks were found with surrounding enhancer histone marks in 1,004 HSEL (Additional file 1: Figure S13A, B). Among these sites, 550 HSEL were poised, and 454 of them were putatively active (Figure 4A). On the other hand, among our 2,000 MSEL, 1,047 coincided with MDAMB231 FAIRE peaks (n = 46,552) with surrounding enhancer histone marks (Figure 4B, Additional file 1: Figure S13C, D). Among them, 512 were poised, and 535 were putatively active (Figure 4B). As *a priori* defined, H3K4me1 marks were more enriched than H3K27Ac in poised enhancers, and H3K27Ac density was much higher than H3K4me1 in active enhancers. However, the mean density of FAIRE peaks in either poised or active enhancers was similar (Figure 4A,B). Overall, regardless of enhancer status, about fifty percent of enhancers were at nucleosome-depleted regions. Although one study reported that H3K27Ac is not enriched in enhancers without DNaseI hypersensitive sites [43], our results suggested that the H3K27Ac mark was not dependent on the presence of nucleosome-depleted regions, as detected by FAIRE. Poised enhancers still retained nucleosome depletion even without H3K27Ac enrichment (Figure 4C).

We investigated this relationship even further by correlating FAIRE signals with the expression levels of genes within a 100 kb window of the signal. When the top 2,000 cell-type specific nucleosome-depleted regions were selected and nearby gene expression levels were calculated, significant correlations were apparent between the nearby gene expression levels and presence of cell type specific FAIRE signals (data not shown).

In order to determine whether the presence of FAIRE peaks in HSEL and MSEL can further increase the apparent specificity of association with cell-type specific gene expression, we again examined gene expression. When the poised and active cell type specific enhancers were further classified according to FAIRE signals, and the expression level differences of nearby genes (+/-100 kb) were assessed, HSEL with FAIRE signals seemed to more strongly associate with nearby cell type specific gene expression levels at both enhancer states (Additional file 1: Figure S14A, C). However, there was no statistically significant difference in gene expression levels near MSEL by the presence of FAIRE signals (Additional file 1: Figure S14B, C). In our analyses, FAIRE signals in enhancers seemed to significantly affect gene expression levels in normal breast epithelial cells, but not in the breast cancer cells. In addition, when all of the FAIRE signals in HMEC (n = 57,489) were intersected with 11 different histone modification marks (i.e. H3K27me3, H2A.Z, H3K9me3, H3K79me2, H3K36me3, H3K20me1, H3K9Ac, H3K4me2, H3K4me3, H3K27Ac, H3K4me1), more than ninety-seven percent of peaks coincided with above histone marks (n = 55,986). Therefore, the regions identified by FAIRE seem to represent a subgroup of regions enriched by various histone marks [44, 45], indicating the importance of histone marks surrounding the FAIRE signals than FAIRE signals themselves.

### Transcription factors in breast epithelial cell type specific enhancers

Nucleosome-depleted regions are known as sites of transcription factor occupation [18, 46]. Therefore, we searched for such motifs, which may be found at FAIRE peaks in HSEL and MSEL by using TRANSFAC and JASPAR [47, 48]. This database also contains motif matrices from best-scoring TF binding sites identified with a ChIP-chip or ChIP-seq fragment. The search was restricted to the response elements of factors that were known to be expressed in these cells [33]. A chi-square test was performed between the groups to measure the enrichment of transcription factor response elements (summarized in a flow diagram, see Additional file 1: Figure S15).

The results of transcription factor motif searches at FAIRE signals in the HSEL and MSEL revealed that several response elements such as TP63, TFCP2, SMAD3, NF1, and EP300 elements were more highly enriched at HSEL than at MSEL (Table 1). On the other hand, FOS, FOXA, and TCF4 were highly enriched in the FAIRE regions at the MSEL, compared to at HSEL. TP63, which is the most significantly enriched motif in HSEL compared to MSEL, is known to act as a sequence-specific DNA binding transcriptional activator or repressor. TP63 plays an essential role in epidermal development and regulates multiple pathways such as BMP and Notch signaling [49, 50]. TP63 has complicated roles in human cancer: some studies reported its tumor suppressor activity in breast and bladder cancers, but it acted as an oncogene in lung cancer cells [51–53]. It may be required in conjunction with TP73/p73 for initiation of p53/TP53 dependent apoptosis in response to genotoxic insult and the presence of activated oncogenes [54, 55]. In order to test whether transcription factors, which were identified by motif searches, physically bind to breast epithelial cell type specific enhancers, we performed site-specific ChIP analyses by using TP63 antibody in both HMEC and MDAMB231 cells. As the motif search suggested, TP63 was enriched in multiple cell type-specific enhancers of HMEC, located in the intron of the *TP73* gene in 1p36.32, gene desert region in 2q13, intron of *SCHIP1* gene in 3q25.33, and gene desert region in 10q24.33. However, at the same enhancer no TP63 binding was detected in MDAMB231 cells; neither enhancer histone marks nor a FAIRE signal was detected in MDAMB231 cells (Additional file 1: Figure S16, Figure S17). The observation that TP63 binding coincided with open chromatin regions marked by enhancer histone modifications, may be related to their relative expression and protein levels and/or to the fact that the respective cell-type specific enhancers were more or less permissive to transcription factor binding due to their chromatin structures. In order to further investigate this, we measured the expression level of *TP63* gene in HMEC and MDAMB231 by performing additional RT-qPCR and re-visiting the data from microarray data (Additional file 1: Figure S18). Although there was a small discrepancy between expression levels among exons we measured, *TP63* gene was expressed at higher levels in HMEC than MDAMB231. We also measured protein levels of TP63 using western blots, and found that TP63 protein level was higher in HMEC than MDAMB231 (Additional file 1: Figure S19). Therefore, our data here cannot distinguish if cell type specific TP63 binding in these enhancers is due to its protein level and/or chromatin accessibility. However, recent studies reported that TP63 binding may require accessible chromatin and/or additional transcription factors that cooperatively associate with DNA [56, 57]. Further investigations on TP63 activity such as its possible ability to penetrate local nucleosome structures as a pioneer protein or recruit other factors will facilitate the understanding of TP63 binding mechanism in breast cancer.

**Table 1:** **Motif enrichment in cell type specific enhancers**

Motif name	Number of motif in HSEL	Number of motif in MSEL	P-value	Database
TP63	116	4	7.36E-30	TRANSFAC
FOS	398	650	2.90E-24	JASPAR
FOS	352	589	3.09E-21	JASPAR
FOS	49	173	6.27E-19	TRANSFAC
FOS	42	183	2.56E-17	TRANSFAC
FOXA1	277	468	8.01E-16	JASPAR
FOS	352	585	5.15E-14	JASPAR
FOS	39	133	9.59E-14	TRANSFAC
FOXA2	339	514	1.92E-12	JASPAR
FOS	32	143	2.92E-12	TRANSFAC
FOXA1	47	124	4.37E-12	TRANSFAC
FOXA1	32	134	6.36E-12	TRANSFAC
FOS	306	524	6.91E-12	JASPAR
TCF4	95	215	2.33E-10	TRANSFAC
FOXA1	49	117	3.29E-10	TRANSFAC
FOXA1	34	127	4.05E-10	TRANSFAC
FOXA2	9	59	1.41E-08	TRANSFAC
FOXA1	40	129	1.48E-08	TRANSFAC
FOXA1	43	89	2.57E-07	TRANSFAC
FOXA1	28	99	2.91E-07	TRANSFAC
FOXI1	232	347	4.49E-07	JASPAR
FOXA1	34	122	4.80E-07	TRANSFAC
FOXF2	128	214	3.00E-06	JASPAR
FOXD1	121	202	6.78E-06	JASPAR
NR4A2	226	326	1.07E-05	JASPAR
FOXA1	38	94	1.53E-04	TRANSFAC
MAF	220	324	2.61E-04	TRANSFAC
FOXQ1	150	221	2.86E-04	JASPAR
FOXO3	221	303	3.23E-04	JASPAR
MYB	110	139	5.55E-04	TRANSFAC
BACH2	11	36	1.08E-03	TRANSFAC
RORA	208	257	3.94E-03	JASPAR
NFATC2	250	320	4.21E-03	JASPAR
TFCP2	223	200	5.15E-03	TRANSFAC
ELK1	64	101	6.46E-03	JASPAR
SMAD3	106	83	6.92E-03	TRANSFAC
NFE2L2	277	344	9.47E-03	JASPAR
HLTF	81	120	9.77E-03	JASPAR
PPARA	69	84	1.18E-02	TRANSFAC
NFYA	110	152	1.57E-02	JASPAR
MYB	78	97	1.76E-02	TRANSFAC
GABPA	337	404	1.80E-02	JASPAR
RORA	176	237	1.80E-02	JASPAR
MYB	73	107	1.83E-02	JASPAR
NF1	90	72	1.95E-02	TRANSFAC
EP300	170	153	2.05E-02	TRANSFAC
RORA	174	207	3.84E-02	JASPAR
ZEB1	25	15	4.47E-02	TRANSFAC

When we further compared the enriched motifs between the poised and active enhancers, besides above factors, NR3C1, NFATC2, FOXQ1, and PBX1 motifs were significantly enriched at active HSEL than poised HSEL. Conversely, VDR, TFAP2A, TFCP2, BHLHE41, and NFIC motifs were more enriched at poised HSEL than active HSEL (Additional file 2: Table S9). Amongst the MSEL, FLI1//EWSR1, GABPA, ELK1, MYB, and NR2F1 were enriched at active MSEL than poised MSE. Conversely, FOXA motifs were significantly enriched in poised MSEL relative to active MSEL (Additional file 2: Table S10).

### Identification of target genes for each cell type specific enhancer

Nearby genes of cell type specific enhancers (i.e. HSEL and MSEL) were studied to identify more specifically those target genes, whose expression levels were regulated mainly by cell type specific enhancers. For each cell type, there were about 1,400 genes (1,418 for HSEL, 1,405 for MSEL) in 100 kb windows around cell type specific enhancers. Among them, 376 genes were found in both windows of cell-type specific enhancers since some cell-type specific enhancers were clustered. In order to identify specific target genes for each cell type specific enhancer, the overlapped genes were excluded for further analysis. Among the genes located near HSEL only (n = 1,042), 59 percent (n = 617) had higher expression levels in HMEC than MDAMB231, whereas 29 percent (n = 303) had higher expression levels in MDAMB231 than HMEC (p < 0.05). Similarly, among the genes located near MSEL only (n = 1,029), 55 percent (n = 567) had higher expression levels in MDAMB231 than HMEC, whereas 33 percent (n = 335) had higher expression level in HMEC than MDAMB231 (p < 0.05) (Additional file 2: Table S11).

**Figure 5:**
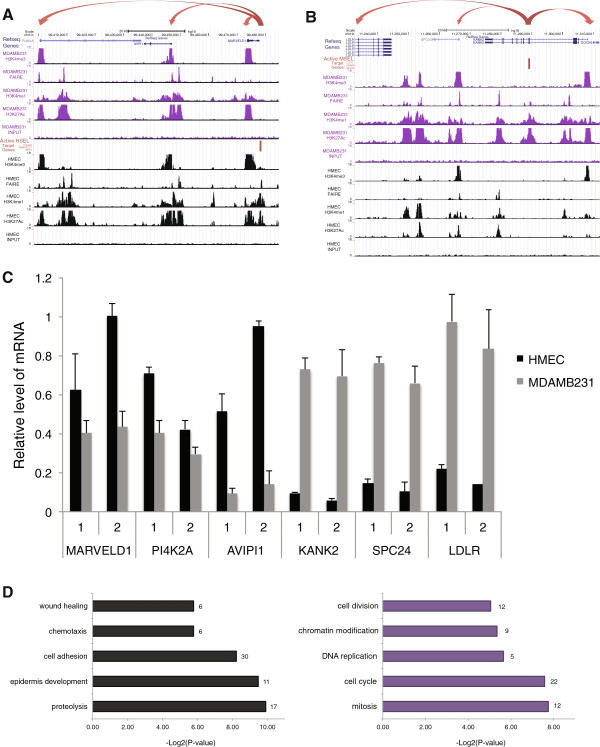
**Identification of target genes, which may be regulated by cell type specific enhancers. (A)** UCSC genome browser screenshot for an example of active HSEL (red bar) and their putative target genes (red arrows) **(B)** UCSC genome browser screenshot for an example of active MSEL (red bar) and their putative target genes (red arrows) **(C)** Quantitative real-time RT-PCR analysis of putative target genes of active HSEL (i.e. *MARVELD1, PI4K2A, AVIPI1)* and active MSEL (i.e. *KANK2, SPC24, LDLR*)*.* Two primer sets located in the exons of each gene were used, and the expression levels were presented relative to *GAPDH* expression. The error bars indicated the standard deviations from triplicate in HMEC (black) and MDAMB231 (grey). **(D)** Gene ontology process categories, which were differentially enriched in the putative target genes of active HSEL (black) and active MSEL (purple). x-axis indicated the -Log2 of p-value, calculated by performing chi-square between groups. The number of genes belonged to each GO category was shown on the right side of the bar.

Furthermore, active enhancers regulated gene expression level more significantly than poised enhancers, and they were engaged with promoters of target genes through DNA looping [39]. Therefore, among cell-type specific enhancers, only active enhancers as defined above were chosen, and their putative specific target genes were matched by using nearby cell type specific genes (within 100 kb). Finally, we identified 316 genes, with higher expression levels in HMEC than MDAMB231 near active HSEL (named as HMEC selected genes). These genes were considered regulated by 323 active HSEL (Additional file 2: Table S12). Similarly, 342 genes were identified with higher expression levels in MDAMB231 than HMEC near active MSEL (named as MDAMB231 selected genes). Thus, 309 active MSEL near these genes likely regulate their expression (Additional file 2: Table S13). As an example, one of the active HSEL, which was located 4.5 kb downstream of the transcription start site of the gene, *MARVELD1* seemed to increase the expression level of *MARVELD1, PI4K2A*, and *AVPI1* genes in HMEC (Figure 5A). Another example was the active MSEL, located in the intron of *KANK2* genes, and it may regulate the gene expression levels of *SPC24*, *LDLR*, and *KANK2* (Figure 5B). In order to verify the expression levels of these genes in both cell types, we performed RT-qPCR by designing two primer sets for each gene (Figure 5C). *MARVELD1, PI4K2A*, and *AVPI1* genes, near the HSEL in 10q24, were expressed higher in HMEC than MDAMB231. Conversely, *SPC24*, *LDLR*, and *KANK2* genes, near the MSEL in 19p13, were expressed at higher levels in MDAMB231 than HMEC. According to our calculations of all enhancer specific gene, up to 8 nearby genes are regulated by one active cell type specific enhancer, and up to 8 different active cell type specific enhancers may regulate one gene.

Lastly, the selected genes, which we identified as putatively regulated by active cell type specific enhancers, were further analyzed by performing gene ontology (GO). When the difference between enrichment of genes in both cell types for each GO biological process category was measured, it was found that there were more genes involved in proteolysis, epidermis development, cell adhesion, chemotaxis, and wound healing in HMEC selected genes than MDAMB231 selected genes (p < 0.02). On the other hand, of the genes that we proposed to be under the control of MDAMB231 specific enhancers, a greater proportion were classified as being involved in mitosis, cell cycle, DNA replication, chromatin modification, and cell division, compared to HMEC selective genes (p ≤ 0.03) (Figure 5D). In order to illustrate relationships among differentially enriched GO process categories of HMEC and MDAMB231 selected genes, a comparison chart was generated using Quick GO [58] (Additional file 1: Figure S20).

Genes, that are involved in differentially enriched GO biological process categories are listed in a Table 2. For example, *LAMC2*, which is involved in epidermis development and cell adhesion, is overexpressed in HMEC than MDAMB231. This gene is translated as a gamma 2 Laminin protein, which belong to a family of extracellular matrix glycoproteins that constitute basement membranes. This gene is reported to be down-regulated in various human cancers including breast cancers [59]. As another example, *SPC24*, which is involved in mitosis, cell cycle, and cell division process, is expressed at higher levels in MDAMB231 than HMEC (Figure 5B). SPC24 acts as a component of NDC80 kinetochore complex, which is required for chromosome segregation and spindle checkpoint activity. This gene is expressed at higher level in grade 3 breast tumors, which are poorly differentiated but highly proliferative [60]. SPC24 protein interacts with BUB1B protein according to the previous study, which identified mitotic protein complexes by using tandem-affinity purification of human epithelial cells arrested in mitosis [61]. *BUB1B* is also one of our MDAMB231 selected genes, regulated by the MSEL in 15q15 (Table 2). *BUB1B*, which is also known as Budding Uninhibited By Benzimidazules 1, encodes a kinase involved in spindle checkpoint function. This protein inhibits the anaphase-promoting complex (APC) and delays the onset of anaphase, which will ensure proper chromosome segregation [62]. Previous studies reported that *BUB1B* expression level is altered in breast tumors, and the encoded protein is mutated in colorectal cancer and breast cancer [63, 64]. BUB1B protein is also known to activate P53 via phosphorylation [65].

**Table 2:** **A list of selected genes belong to differentially enriched gene ontology (GO) biological process**

GO process	Genes
Proteolysis	*ADAMTS3, CPA4, CTSB, CTSC, HTRA1, KLK10, KLK5, KLK6, KLK7, KLK8, MMP2, PREP, PRSS12, PRSS8, PYCARD, ST14, ZFP90*
Epidermis development	*ATP2A2, COL17A1, GJB5, KLK5, KLK7, KRT5, LAMC2, PTHLH, S100A7, SPRR1A, SPRR1B*
Cell adhesion	*ATP2A2, BOC, CDH3, CELSR2, CLDN1, CLSTN1, CTNND1, DDR1, DSC2, DSC3, DST, F11R, FBLIM1, FLRT2, IGFBP7, ITGB8, LAMC2, MTSS1, NID1, NRP2, NTM, PTK7, PTPRF, SDK2, SIRPA, SPOCK1, SSPN, SSX2IP, TRIP6, ZYX*
Chemotaxis	*CMTM6, CXCL14, IL8, PTAFR, S100A8, S100A9*
Wound healing	*CDH3, IL1A, IL1B, SDC1, SERPINB2, TPM1*
Mitosis	*ANAPC1, BIRC5, BUB1B, CCNF, CEP55, KIF2C, NCAPG2, SPC24, TPX2, TUBB, TUBB2A, UBE2C*
Cell cycle	*ANAPC1, BIRC5, BUB1B, CCNF, CDKN1B, CDKN3, CEP55, DDX11, DUSP1, GSG2, ING1, ITGAE, KIF2C, LLGL2, NCAPG2, PARD3B, PIN1, RPS6KB1, SPC24, STK11, TPX2, UBE2C*
DNA replication	*MCM10, POLD1, POLG2, RRM2, TK1*
Chromatin modification	*ASF1B, ASXL1, EZH1, GSG2, H2AFY, MLL, NSD1, TAF5, WHSC1*
Cell division	*ANAPC1, BIRC5, BUB1B, CCNF, CEP55, KIF2C, LLGL2, NCAPG2, PARD3B, SPC24, TPX2, UBE2C*

Next, we tested whether HMEC selected genes, which may be regulated by HSEL, were involved in different biological processes from randomly chosen overexpressed genes in HMEC (n = 316). Interestingly, the gene ontology categories found above (i.e. proteolysis, epidermis development, and cell adhesion) were not differentially enriched between HMEC selected genes and random data set (Additional file 2: Table S14). When we compared MDAMB31 selected genes and randomly chosen overexpressed genes in MDAMB231 (n = 342), the gene ontology categories found above (i.e. mitosis, cell cycle, DNA replication, chromatin modification, and cell division) were not differentially enriched between MDAMB231 selected genes and random data set (Additional file 2: Table S15). As additional control datasets, we further tested whether HMEC selected genes and randomly chosen overexpressed genes in MDAMB231 were involved in different biological processes. Interestingly, GO process categories, differently enriched between HMEC and MDAMB231 selected genes (e.g. mitosis, cell cycle, cell division, cell adhesion, epidermis development) were also differentially enriched in this comparison (Additional file 2: Table S16). Conversely, same GO process categories were found to be differentially enriched from comparison between MDAMB231 selected genes and randomly chosen overexpressed genes in HMEC (Additional file 2: Table S17). Therefore, we propose that genes located proximal to HSEL or MSEL seem to be involved in similar biological processes (e.g. mitosis, cell cycle, cell division, cell adhesion, epidermis development) defined by differentially expression. In summary, we did not find any distinct biological process category that was enriched in genes located proximal to HSEL or MSEL, compared to the differentially expressed genes.

## Conclusions

Recent ENCODE data reveal that the average number of enhancers interacting with a TSS was 3.9, and the average number of TSSs interacting with a distal element was 2.5 in human cells [66]. Therefore, it is likely that a large number of chromosomal contacts and interactions are orchestrated by the three-dimensional organization of the nucleus. In the spatial genome organization, intra- and inter-chromosomal interactions are mediated by nuclear components such as transcription and replication machinery, polycomb bodies, and contacts with the lamina [67–69]. In the study from the Dekker lab, when the contact probability was estimated across all chromosomes in human cells, the contact probability decreased as the genomic distance increased, showing a power-law behavior between genomic distance 500 kb and 7 Mb with an average exponent α ∼ 1.08. This observation led to the suggestion that the organization of chromatin packing was consistent with the behavior of a fractal globule, a knot-free, polymer conformation [70].

Consequently, the proximity of genes to enhancers on linear DNA, which controls their expression, has long been thought to be the main factor of transcriptional regulation via enhancers, called cis interactions. On the other hand, trans interactions between enhancers and genes on different chromosomes are possible to regulate gene expression, but less frequent [70]. Methods that define interactions between DNA regions and gene expression include chromatin conformation capture (3C) and its derivative methods (3C-seq, 4C-seq, 5C-seq, ChIA-PET and HiC-seq) [71, 72], expressed quantitative trait loci (eQTLs) [45], the use of transcription activator-like effector nucleases (TALEN or CRISPR) [73], and knock-out mouse models [74, 75]. However, methods employing the capture of looping interactions by cross-linking chromatin are technically demanding for cis interactions since the background signals of random collisions (thus false positives) are high. Therefore, it is almost impossible to distinguish the interaction frequency of looping between a gene and sites located within 50 kb away from no looping [76]. Additionally, eQTL analyses, TALEN, and transgenic mouse modeling cannot inform whether the interactions are direct or indirect.

Here we evaluated cis interactions between enhancers defined by histone modification and nearby genes in normal breast epithelial cells (HMEC) and breast cancer epithelial cells (MDAMB231). We showed that enhancers affected the expression levels of nearby genes up to 750 kb away on average, but the effect diminished as the distance increased. Enhancer status defined by histone marks correlated with the expression levels of nearby genes. Nucleosome-depletion, marked by FAIRE signals was present in fifty percent of both poised and active enhancers. By performing a motif search in enhancers and ChIP assays, we revealed transcription factors (e.g. TP63), which may be involved in regulating breast epithelial enhancer-mediated gene expression. We also identified putative target genes of the active cell type specific enhancers by genomic distance (i.e. within 100 kb) with cell type specific gene expression. The putative target genes of cell type specific enhancers in normal breast epithelial cells (HSEL) were enriched in proteolysis, epidermis development, and cell adhesion biological process. On the other hand, more genes potentially regulated by breast cancer cell type specific enhancers (MSEL) were involved in mitosis, cell cycle, DNA replication, chromatin modification, and cell division process. When we further compared expression levels of these genes in normal breast and invasive breast carcinoma tissues using the cancer genome atlas (TCGA) breast cancer data [77, 78], we found that 60 genes of MDAMB231 selected genes belonged to top 10 percent overexpressed genes in breast tumors than normal breast tissues, and 53 genes of HMEC selected genes belonged to top 10 percent under-expressed genes (Additional file 2: Table S18, Table S19). The list of these genes includes not only a large number of known breast cancer genes (e.g. *CDH1, CDH3, BMP4, MTAP, CDKN2B*), revealed by previous studies using breast tumor tissues and breast cancer cell lines [79, 80] but also novel genes. The list of more than 600 putative target genes of more than 600 selected cell type specific enhancers and identified transcription factors will facilitate understanding of epigenetic regulation in breast biology as well as cancer etiology.

## Methods

### Cell Culture

HMEC cells were obtained from Lonza (Lonza, Walkersville, MD) and cultured under recommended conditions. MDAMB231 cells were obtained from American Type Culture Collection (ATCC, Manassas, VA). MDAMB231 cells were cultured in DMEM with 5% FBS.

### FAIRE-seq library construction and sequencing

FAIRE assays were performed as described [42], with a number of modifications. Briefly, the method was as follows: (1) intact cells were crosslinked (1% formaldehyde in PBS); (2) nuclei were extracted from cells and re-suspended in SDS lysis buffer; (3) chromatin DNA was fragmented by sonication; (4) FAIRE DNA samples and reverse-crosslinked input DNA were purified by phenol-chloroform extraction. Two independent libraries were constructed for each sample by using bar-coded adapters. Each library was PCR amplified and confirmed by quantitative real-time PCR (qPCR). Single-end DNA sequencing (Illumina Hi-Seq 50 cycles) was performed at the USC Epigenome center. After passing through the pre-alignment QC pipeline, which checked for things such as adapter and poly-A contamination, reads were aligned with BWA 0.6.1 using the default parameters. The reference genome used was GRCh37/HG19. Two independent assays per condition were analyzed separately, and then combined for further analyses. HMEC FAIRE-seq data were deposited in the NCBI GEO under accession number, GSE46074 [45]. MDAMB231 FAIRE-seq data were deposited in the NCBI GEO number accession number, GSE49651.

### Identification of FAIRE-seq peaks

After removing PCR artifacts and duplicates, each bam file was filtered using a quality filter score of 30 by using Samtools [81]. FindPeaks 4.0 software [82] was applied in order to identify FAIRE-seq peaks. A triangle-based distribution with a median length of 150 bp and 99.0% confidence interval for peak pairs, which were unequal between sample and input, were used. After peak identification, we calculated a p-value for each peak between sample and input, and significant peaks were used for further analysis.

### Chromatin Immunoprecipitation library construction and sequencing

HMEC Histone modification ChIP-seq data (H3K4me1, me3, and H3K27Ac) were obtained from accession number [GSE29611] through the NCBI Gene Expression Omnibus portal. ChIP assays were performed in MDAMB231 as previously described [83]. Antibodies used were anti-H3K4me1 (ab8895) (Abcam, Cambridge, MA), anti-H3K27Ac (ab4729) (Abcam, Cambridge, MA), anti-H3K4me3 (04-745) (Millipore Corp., Billerica, MA), anti-P63 (39739) (Active Motif, Carlsbad, CA), and normal rabbit IgG (sc-2027) (Santa Cruz Biotechnology, Santa Cruz, CA). qPCR were performed on ChIP-isolated and input DNA by using KAPA SYBR FAST qPCR Kits (Kapa Biosystems, Woburn, MA) with primers, listed in Additional file 2: Table S20.

### ChIP-seq library construction and sequencing

Library was constructed and sequenced as previously described above in the FAIRE-seq section. Two independent assays per condition were analyzed separately (Additional file 2: Table S2), and then the data were combined for further analyses. MDAMB231 ChIP-seq data were deposited in the NCBI GEO number accession number, GSE49651.

### Identification of ChIP-seq peaks

Each bam file was filtered using quality filter score 30 after removing PCR artifacts and duplicates by using Samtools [81]. To identify enriched regions of histone modifications (i.e. H3K4me1, H3K4me3, H3K27Ac) against input, MACS software on histone ChIP-seq data was applied with default settings [84]. For HMEC FAIRE-seq peaks overlapping with ChIP-seq peaks analysis, ChIP-seq peaks identified by Scripture [85] for histone marks, H3K27me3, H2A.Z, H3K9me3, H3K79me2, H3K36me3, H3K20me1, H3K9Ac, and H3K4me2, from ENCODE were used [66].

### Cell type specific enhancer loci identification and states

Differently enriched H3K4me1 sites (i.e. HSEL and MSEL) were identified from normalized HMEC and MDAMB231 H3K4me1 ChIP-seq tags by using findPeaks from HOMER ( http://homer.salk.edu/homer/) [20]: two ChIP samples (HMEC H3K4me1 and MDAMB231 H3K4me1) were run as ‘treatment’ and ‘control’ in order to identify significant differences between the samples. To avoid from detecting false positive peaks, 0.10% false discovery rate (FDR) was used as a cut off. In order not to be biased by the different yields of identified peaks in the two cell types, the top 2,000 H3K4me1 sites were selected for each of the HMEC specific enhancer loci (HSEL) and MDAMB231 specific enhancer loci (MSEL). Identified enhancer loci were plotted in the heatmaps and line graphs, which were generated by using seqMINER software [40]. Poised and active enhancers were categorized by using K-means linear clustering (n = 2) with H3K27Ac ChIP-seq tags.

### Shared enhancer loci identification

Shared enhancers were identified from normalized HMEC and MDAMB231 H3K4me1 ChIP-seq tags and peaks by using getDifferentialPeaks from HOMER ( http://homer.salk.edu/homer/) [20]. 2,000 shared enhancers, which have similar ChIP-seq tag density in both cell types were selected for further analysis as cell type specific enhancers were studied.

### Annotation and comparison between HMEC and MDAMB231 cells

Identified peaks were analyzed by using mergePeaks and annotatePeaks from HOMER ( http://homer.salk.edu/homer/) [20]. Annotated positions for promoters, exons, introns, intergenic regions, and other features were based on RefSeq transcripts and repeat annotations from University of California, Santa Cruz. Cell type specific enhancer loci were visualized in genome using Circos software [86].

### Motif discovery and enrichment measurements

In order to find regulatory motifs in enhancers, sets of position weight matrices (PWMs) were used from FIMO and TRANSFAC/Genome Trax [47, 48, 87]. FIMO analysis was performed using the motif database, called JASPAR CORE 2009 vertebrates, downloaded from the MEME suite ( http://tools.genouest.org/tools/meme/meme-download.html) [87]. P-value for output threshold utilized for FIMO was 1e-4. Predicted ChIP-seq TFBS analysis, predicted TFBS in DNase hypersensitivity regions, and TRANSFAC experimentally verified TFBS data from Genome Trax were obtained. This database contains motif matrices from best-scoring TF binding sites identified with a ChIP-chip or ChIP-seq fragment. The enrichment of transcription motifs in enhancer regions was calculated by performing chi-square test between groups (P < 0.05).

### Gene expression analysis between HMEC and MDAMB231 cells

Gene expression data for HMEC and MDAMB231 cells, which were detected in the affymetrix HG-U133 plus2 microarrays, were obtained from the accession number [GSE33167] [33]. Gene expression levels for both cells as well as p-values were processed by using GEO2R [88]. Adjusted p-value cut off 0.05 was applied to identify HMEC and MDAMB231 overexpressed genes (3,174 genes for HMEC, 2,670 genes for MDAMB231). Log fold change of gene expression level between cell types was graphed using box plots in R [89]. Log fold change value was used in order to identify top 300 overexpressed genes in each cell type, which were about top 10 percent. The enrichment of genes in each category was calculated by performing chi-square test between groups. As control genes, 300 genes were randomly selected among 6,902 genes, which contain 3,174 overexpressed genes in HMEC, 2,670 overexpressed genes in MDAMB231, and 1,058 genes significantly expressed in both cells.

### Quantitative real time RT-PCR

Total RNA from HMEC and MDAMB231 cells were isolated using Aurum Total RNA Mini Kit (Bio-Rad, Hercules, CA). Isolated RNAs were reverse transcribed to cDNA by using the qScript cDNA Synthesis Kit (Quanta Biosciences, Gaithersburg, MD). Two primer sets for each selected gene were designed (Additional file 2: Table S20), and quantitative real-time PCR was performed by using KAPA SYBR FAST qPCR Kits (Kapa Biosystems, Woburn, MA). The raw expression values were normalized to *GAPDH* mRNA expression in each cell type.

### Western blot analysis

Whole cell extracts were prepared from HMEC and MDAMB231 cells using SDS Lysis buffer supplemented with protease inhibitor (Sigma-Aldrich, St. Louis, MO). Equal amounts of protein from whole cell extracts were separated by running on gradient polyacrylamide gels (Bio-Rad, Hercules, CA), and transferred to Amersham Hybond polyvinylidene difluoride (PVDF) membranes (GE Healthcare, Buckinghamshire, UK). These blots were first incubated with either a 1:2000 of anti-P63 (39739) (Active Motif, Carlsbad, CA) or anti-beta-tubulin (MAB3408) (Millipore Corp., Billerica, MA) for overnight, and followed by incubation with their corresponding secondary antibodies, anti rabbit-HRP-conjugated antibody (diluted 1:2500) (sc-2030) and anti mouse-HRP-conjugated antibody (diluted 1:4000) (sc-2031) (Santa Cruz, Dallas, Texas). Proteins were visualized using SuperSignal West Pico Chemiluminescent Substrate (Pierce, Rockford, IL, USA) and ChemiDoc XRS + Imaging System with Image Lab (Bio-Rad, Hercules, CA).

### Gene ontology

Gene ontology analysis was performed by using GEO2R [88]. Same number of genes as samples was randomly selected for controls, and the gene ontology of these genes was also annotated. The enrichment of genes in each gene ontology category was calculated by performing chi-square test between groups. In order to visualize GO data, GO Slims and GO Term Comparison tool from Quick GO [58] was used.

### Comparison between the putative target genes of cell type specific enhancers and differentially expressed genes in breast tumor tissues

By using Oncomine database, released in March 2014, the dataset from the cancer genome atlas (TCGA) breast cancer studies was obtained [77, 78]. The top 10 percent over and under-expressed genes (n = 2,039 genes) detected in the comparison between invasive breast carcinoma and normal tissues were intersected with our putative target genes.

## Electronic supplementary material

Additional file 1: **Figure S1.** ChIP-seq data overlap between HMEC and MDAMB231; **Figure S2.** Genomic distribution of HMEC specific enhancer loci (HSEL); **Figure S3.** Genomic distribution of MDAMB231 specific enhancer loci (MSEL); **Figure S4.** The expression value of nearby genes of HSEL and MSEL; **Figure S5.** Gene expression level for genes with cell type specific enhancers; **Figure S6.** Nearby gene expression barplot of cell type specific enhancers; **Figure S7.** The expression values of all genes in HMEC and MDAMB231; **Figure S8.** The expression levels of genes, which were not within windows of cell type specific enhancer loci; **Figure S9.** Shared enhancer loci identification in breast epithelial cells (HMEC and MDAMB231); **Figure S10.** The expression levels of genes nearby shared enhancers**; Figure S11.** Nearby gene expression boxplot of cell type specific enhancers; **Figure S12.** The expression levels of genes at each distance interval from enhancer loci**; Figure S13.** Poised and active cell type specific enhancers with FAIRE signals; **Figure S14.** Nearby gene expression boxplot for poised and active cell type specific enhancers and FAIRE signal; **Figure S15.** Workflow diagram of transcription factor motif search between enhancer groups; **Figure S16.** TP63 binding in breast epithelial cell type specific enhancers; **Figure S17.** Validation of TP63 binding in breast epithelial cell type specific enhancers in HMEC; **Figure S18.** Expression level of *TP63* gene in breast epithelial cells; **Figure S19.** Western blot analysis of TP63**; Figure S20.** A comparison chart for differentially enriched gene ontology (GO) biological processes. (PDF 2 MB)

Additional file 2: **Table S1.** ChIP-seq and FAIRE-seq peak statistics; **Table S2.** ChIP-seq and FAIRE-seq peak statistics for replicates; **Table S3.** Overlapped ChIP-seq peak statistics for replicates; **Table S4.** HMEC Specific Enhancer Loci coordinates and status; **Table S5.** MDAMB231 Specific Enhancer Loci coordinates and status; **Table S6.** Expression analyses on nearby genes of cell type specific enhancer and shared enhancer; **Table S7.** Shared Enhancer Loci coordinates; **Table S8.** Expression analyses on genes at each distance interval of cell type specific enhancer and shared enhancer; **Table S9.** Motif enrichment in poised HSEL and active HSEL; **Table S10.** Motif enrichment in poised MSEL and active MSEL; **Table S11.** Number of nearby genes (100 kb) for unique HSEL and MSEL in each expression level category; **Table S12.** Active HSEL and their putative target genes; **Table S13.** Active MSEL and their putative target genes; **Table S14.** Gene Ontology in process comparison between HMEC selected genes and randomly selected overexpressed genes in HMEC (n = 316); **Table S15.** Gene Ontology in process comparison between MDAMB231 selected genes and randomly selected overexpressed genes in MDAMB231 (n = 342); **Table S16.** Gene Ontology in process comparison between HMEC selected genes and randomly selected overexpressed genes in MDAMB231 (n = 342); **Table S17.** Gene Ontology in Process comparison between MDAMB231 selected genes and randomly selected overexpressed genes in HMEC (n = 316); **Table S18.** List of the selected MDAMB231 genes found in top 10 percent overexpressed genes in breast tumors; **Table S19.** List of the selected HMEC genes found in top 10 percent underexpressed genes in breast tumors; **Table S20.** Oligonucleotide sequences used for ChIP-qPCR and RT-qPCR. (XLS 657 KB)

## Abbreviations

BCa: Breast cancer
H3K4: Lysine 4 of histone 3
me1: Monomethyl
me2: Dimethyl
me3: Trimethyl
H3K9ac: Acetylated lysine 9 of histone 3
H3K14ac: Acetylated lysine 14 of histone 3
H3K27ac: Acetylated lysine 27 of histone 3
ChIP: Chromatin immunoprecipitation
FAIRE: Formaldehyde Assisted Isolation of Regulatory Elements
TSS: Transcription Start Site
TTS: Transcription Termination Site
HSEL: HMEC specific enhancer loci
MSEL: MDAMB231 specific enhancer loci.
