# Supplementary material for: Nucleosome positioning and histone modifications define relationships between regulatory elements and nearby gene expression in breast epithelial cells
Source: BMC Genomics. 2014 May 2;15(1):331. doi: 10.1186/1471-2164-15-331 (PMC4035062; doi:10.1186/1471-2164-15-331)
Supplement: Supplementary file 1 — Additional file 1: Figure S1. ChIP-seq data overlap between HMEC and MDAMB231; Figure S2. Genomic distribution of HMEC specific enhancer loci (HSEL); Figure S3. Genomic distribution of MDAMB231 specific enhancer loci (MSEL); Figure S4. The expression value of nearby genes of HSEL and MSEL; Figure S5. Gene expression level for genes with cell type specific enhancers; Figure S6. Nearby gene expression barplot of cell type specific enhancers; Figure S7. The expression values of all genes in HMEC and MDAMB231; Figure S8. The expression levels of genes, which were not within windows of cell type specific enhancer loci; Figure S9. Shared enhancer loci identification in breast epithelial cells (HMEC and MDAMB231); Figure S10. The expression levels of genes nearby shared enhancers; Figure S11. Nearby gene expression boxplot of cell type specific enhancers; Figure S12. The expression levels of genes at each distance interval from enhancer loci; Figure S13. Poised and active cell type specific enhancers with FAIRE signals; Figure S14. Nearby gene expression boxplot for poised and active cell type specific enhancers and FAIRE signal; Figure S15. Workflow diagram of transcription factor motif search between enhancer groups; Figure S16. TP63 binding in breast epithelial cell type specific enhancers; Figure S17. Validation of TP63 binding in breast epithelial cell type specific enhancers in HMEC; Figure S18. Expression level of TP63 gene in breast epithelial cells; Figure S19. Western blot analysis of TP63; Figure S20. A comparison chart for differentially enriched gene ontology (GO) biological processes. (PDF 2 MB) [file 12864_2013_6033_MOESM1_ESM.pdf]

Additional file 1: Supplemental Figures

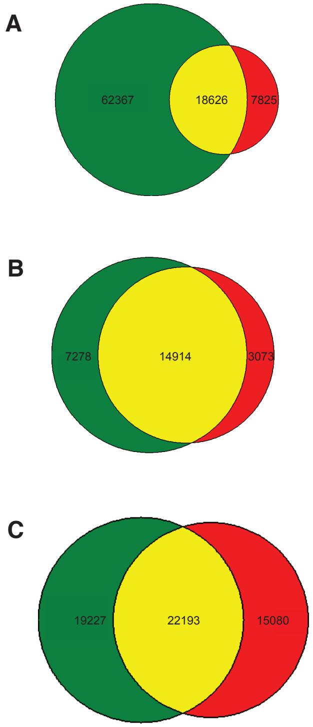

Figure S1. ChIP-seq data overlap between HMEC and MDAMB231

Venn diagram of histone marks ChIP-seq peaks from HMEC (green) and MDAMB231 (red) with overlapped regions (A) H3K4me1 (B) H3K4me3 (C) H3K27Ac

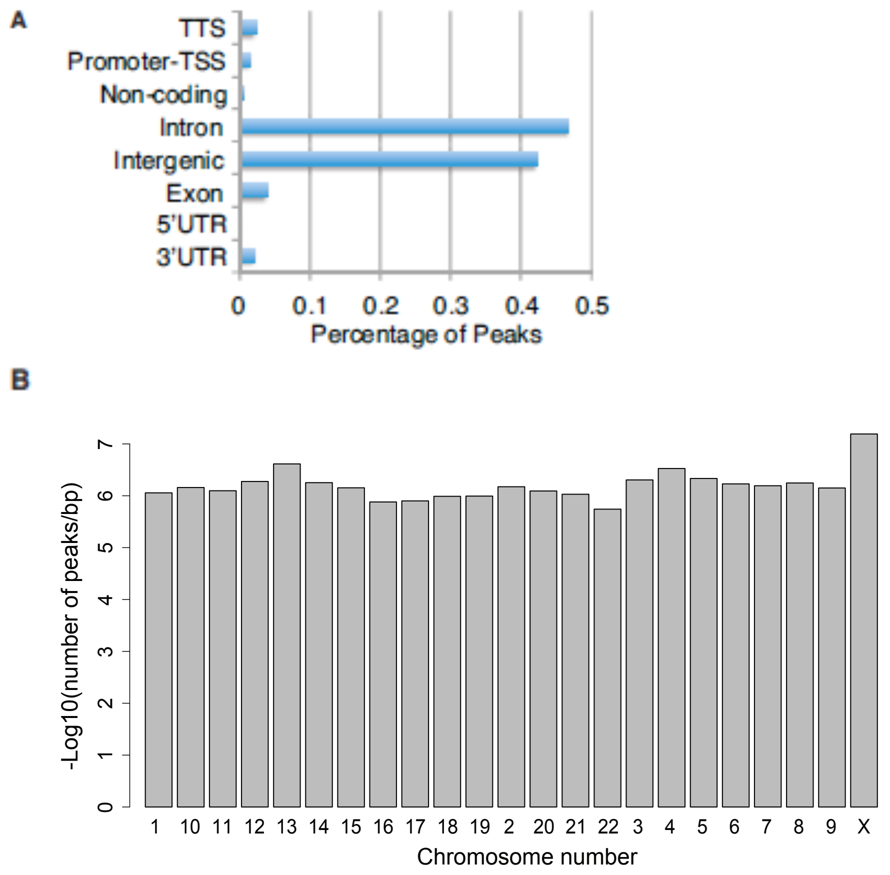

Figure S2. Genomic distribution of HMEC specific enhancer loci (HSEL)

(A) Genomic distribution of the HSEL. Genomic regions were categorized to transcription termination site (TTS) (-100bp of TTS to +1kb of TTS), promoter-transcription start site (TSS) (-1kb of TSS to +100bp), non-coding exon, intron, intergenic, exon, 5'UTR, and 3'UTR (B) Distribution of the HSEL from chr1 to chrX. Number of peaks in each chromosome was normalized to chromosome length, and –Log10 (number of peaks/bp) was shown as y-axis.

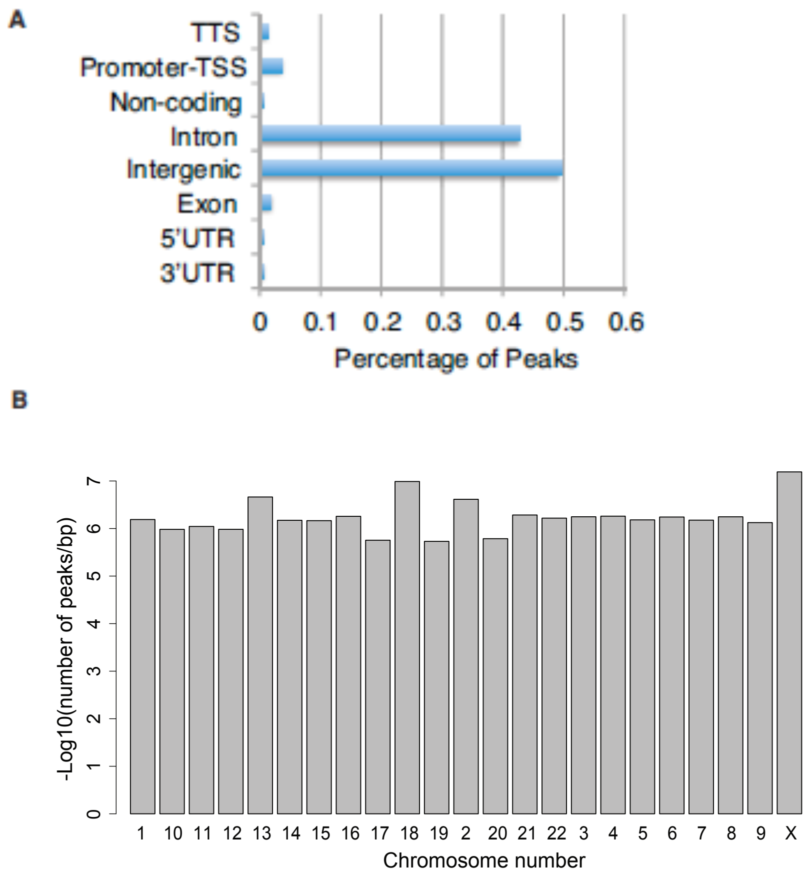

Figure S3. Genomic distribution of MDAMB231 specific enhancer loci (MSEL)

(A) Genomic distribution of the MSEL. Genomic regions were categorized to transcription termination site (TTS) (-100bp of TTS to +1kb of TTS), promoter-transcription start site (TSS) (-1kb of TSS to +100bp), non-coding exon, intron, intergenic, exon, 5'UTR, and 3'UTR (B) Distribution of the MSEL from chr1 to chrX. Number of peaks in each chromosome was normalized to chromosome length, and –Log10 (number of peaks/bp) was shown as y-axis.

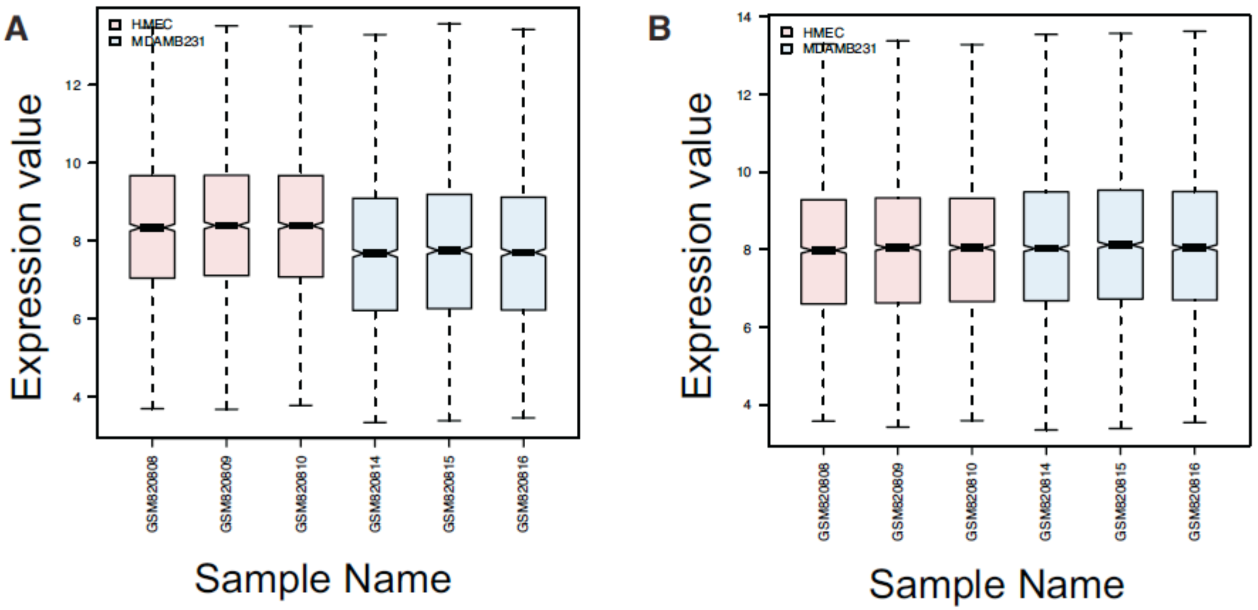

Figure S4. The expression value of nearby genes of HSEL and MSEL

(A) Boxplot of expression value for the genes with HSEL (B) Boxplot of expression value for the genes with MSEL

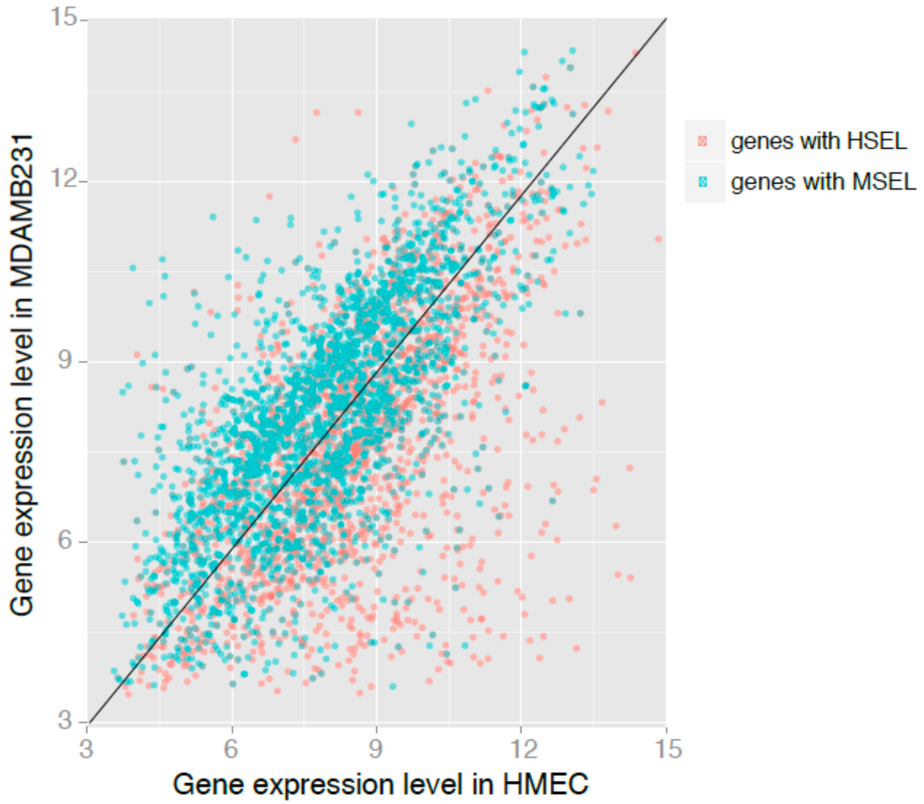

Figure S5. Gene expression level for genes with cell type specific enhancers

Gene expression level in HMEC (x-axis) and MDAMB231 (y-axis) for genes with HSEL (red) and genes with MSEL (blue)

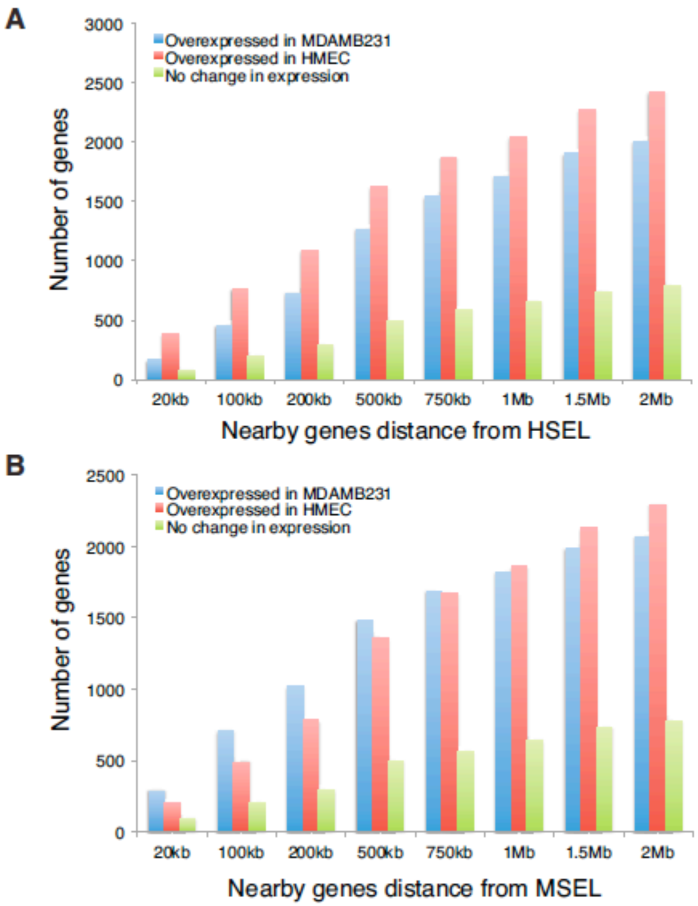

Figure S6. Nearby gene expression barplot of cell type specific enhancers

Nearby genes of cell type specific enhancer loci (HSEL(A), MSEL (B)) were categorized to three groups; overexpressed genes in MDAMB231 (blue), overexpressed genes in HMEC (red), and no change in gene expression (green), and number of genes for each category was graphed. Eight different window sizes were used for the nearby gene distance from cell type specific enhancer loci: from 20kb windows of cell type specific enhancer loci (+-20kb of HSEL/MSEL) up to 2MB windows (+-2MB of HSEL/MSEL).

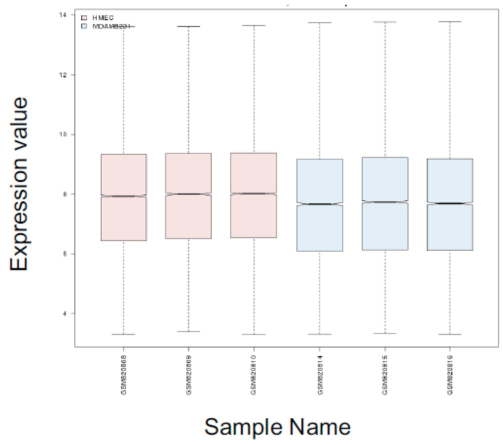

Figure S7. The expression values of all genes in HMEC and MDAMB231

Boxplot of expression values for all of genes expressed in HMEC (pink) and MDAMB231 (blue)

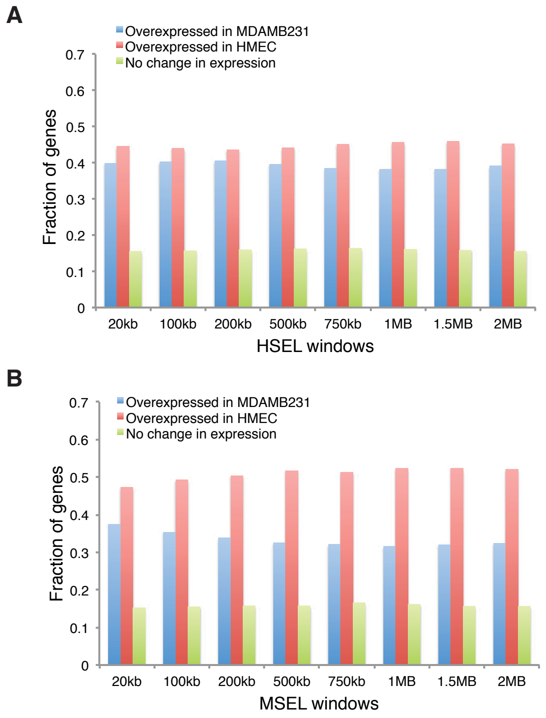

Figure S8. The expression levels of genes, which were not within windows of cell type specific enhancer loci

Genes without cell type specific enhancer (genes were not within windows of cell type specific enhancers) were categorized to three groups; overexpressed genes in MDAMB231 (blue), overexpressed genes in HMEC (red), and no change in gene expression (green), and fraction of genes for each category was graphed. Eight different window sizes were used for the nearby gene distance from cell type specific enhancer loci: from 20kb windows of cell type specific enhancer loci (+-20kb of HSEL/MSEL) up to 2MB windows (+-2MB of HSEL/MSEL). Genes without HMEC specific enhancer loci were plotted in (A), and genes without MDAMB231 specific enhancer loci were plotted in (B).

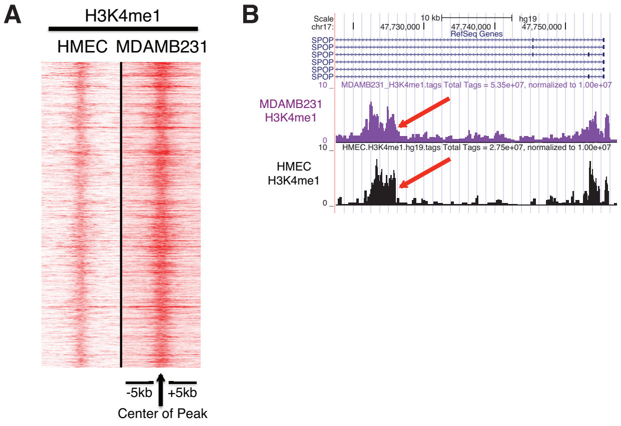

Figure S9. Shared enhancer loci identification in breast epithelial cells (HMEC and MDAMB231)

(A) H3K4me1 ChIP-seq tags from both cells at the center of shared enhancer lcoi were graphed in the heatmap (red: higher density) (B) An example of the shared enhancer loci located in the intron of the *SPOP* gene (red arrow).

A

B

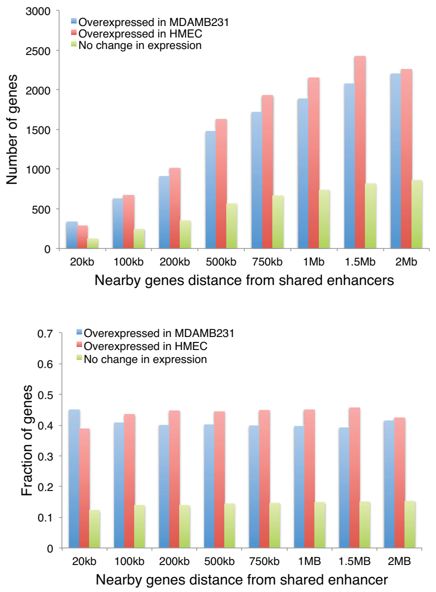

Figure S10. The expression levels of genes nearby shared enhancers

Nearby genes of shared enhancers were categorized to three groups; overexpressed genes in MDAMB231 (blue), overexpressed genes in HMEC (red), and no change in gene expression (green). The number of genes for each category (A) and the fraction of genes for each category (B) were graphed. Eight different window sizes were used for the nearby gene distance from shared enhancers: from 20kb windows of shared enhancer loci (+-20kb of shared enhancer loci) up to 2MB windows (+-2MB of shared enhancer loci).

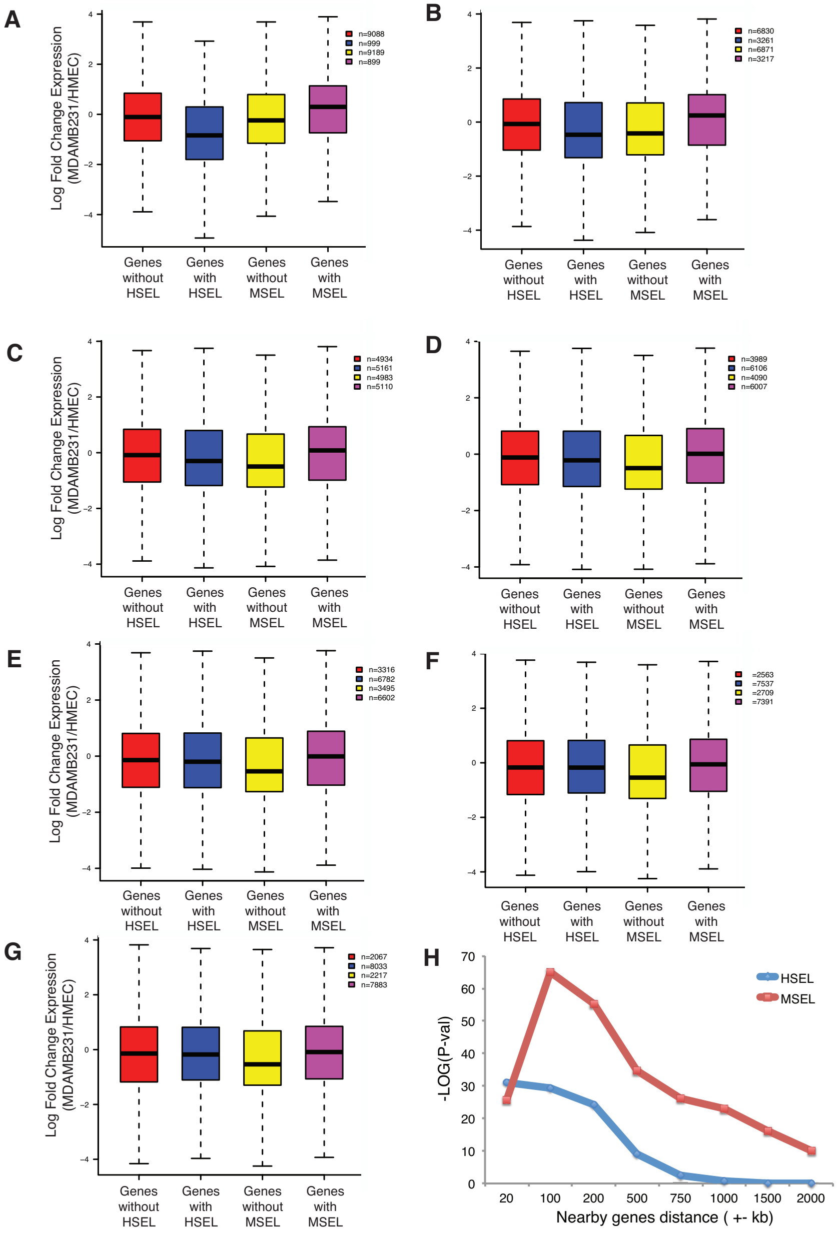
A
Log Fold Change Expression (MDAMB231/HMEC)
n=9088
n=999
n=9189
n=899
Genes without HSEL
Genes with HSEL
Genes without MSEL
Genes with MSEL
B
n=6830
n=3261
n=6871
n=3217
C
n=4934
n=5161
n=4983
n=5110
D
n=3989
n=6106
n=4090
n=6007
E
n=3316
n=6782
n=3495
n=6602
F
=2563
=7537
=2709
=7391
G
n=2067
n=8033
n=2217
n=7883
H
-LOG(P-val)
HSEL
MSEL
Nearby genes distance (+- kb)
20
100
200
500
750
1000
1500
2000

Figure S11. Nearby gene expression boxplot of cell type specific enhancers

Log fold change of nearby gene expression boxplot for the HSEL and MSEL. (A) nearby gene distance in windows of 20kb (B) nearby gene distance in windows of 200kb (C) nearby gene distance in windows of 500kb (D) nearby gene distance in windows of 750kb (E) nearby gene distance in windows of 1Mb (F) nearby gene distance in windows of 1.5Mb (G) nearby gene distance in windows of 2Mb (H) The distribution of significance level for log fold change of nearby gene expression between groups (genes with cell type specific enhancer and genes without cell type specific enhancer) at different windows of nearby gene distance (from 20kb windows of cell type specific enhancers to 2Mb windows of cell type specific enhancers): –LOG(p-value) was plotted in the y-axis for significance level.

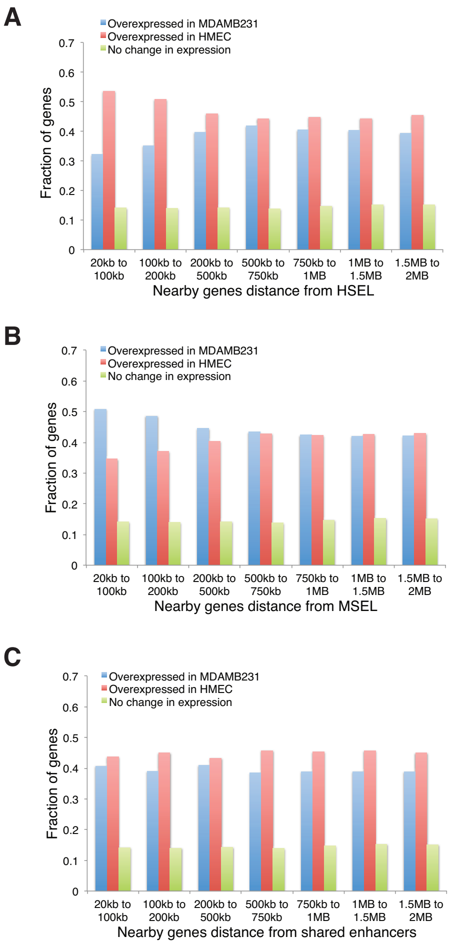

Figure S12. The expression levels of genes at each distance interval from enhancer loci

Nearby genes of HSEL (A), MSEL (B), and shared enhancers (C) were categorized to three groups; overexpressed genes in MDAMB231 (blue), overexpressed genes in HMEC (red), and no change in gene expression (green). The fraction of genes for each category was graphed. Here, unlike previous figures, only genes at each distance interval from window size 20kb to 2MB of enhancer loci were plotted.

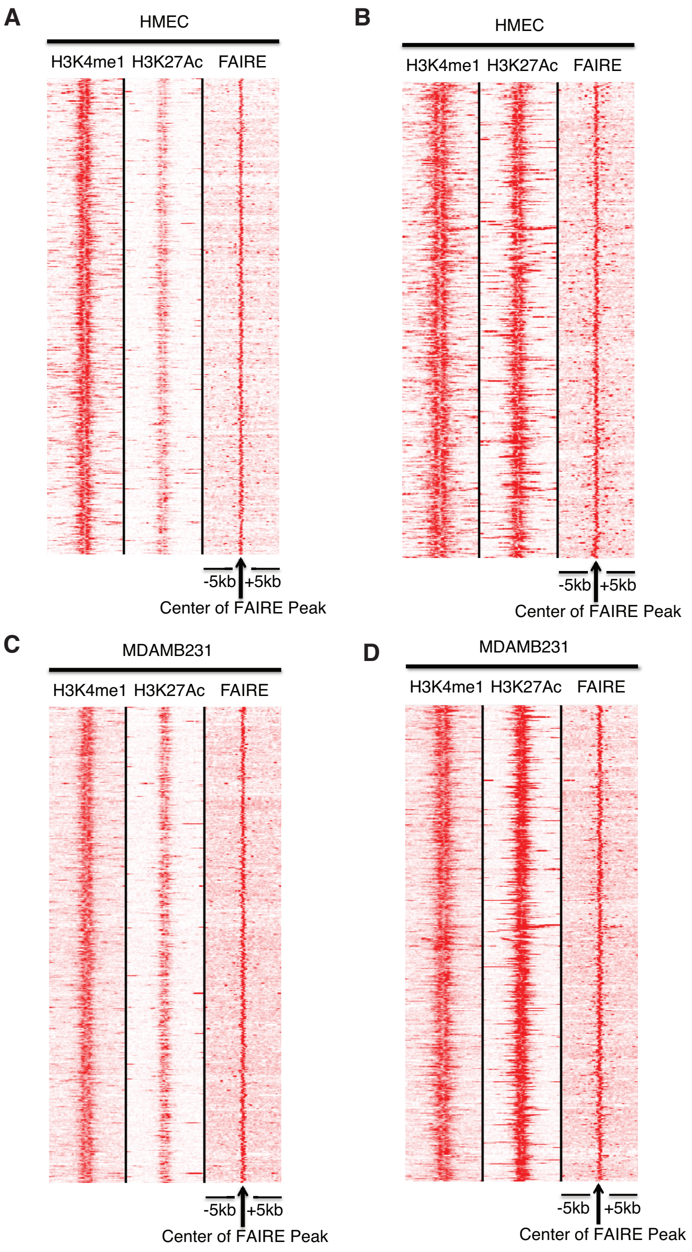
A
HMEC
H3K4me1 H3K27Ac FAIRE
-5kb +5kb
Center of FAIRE Peak
B
HMEC
H3K4me1 H3K27Ac FAIRE
-5kb +5kb
Center of FAIRE Peak
C
MDAMB231
H3K4me1 H3K27Ac FAIRE
-5kb +5kb
Center of FAIRE Peak
D
MDAMB231
H3K4me1 H3K27Ac FAIRE
-5kb +5kb
Center of FAIRE Peak

Figure S13. Poised and active cell type specific enhancers with FAIRE signals

H3K27Ac ChIP-seq and FAIRE-seq tags from HMEC at the poised (A) and active (B) HSEL which were intersected with HMEC FAIRE signals were graphed in the heatmap (red: higher density). H3K4me1, H3K27Ac ChIP-seq and FAIRE-seq tags from MDAMB231 at the poised (C) and active (D) MSEL which were intersected with MDAMB23 FAIRE signals were graphed in the heatmap.

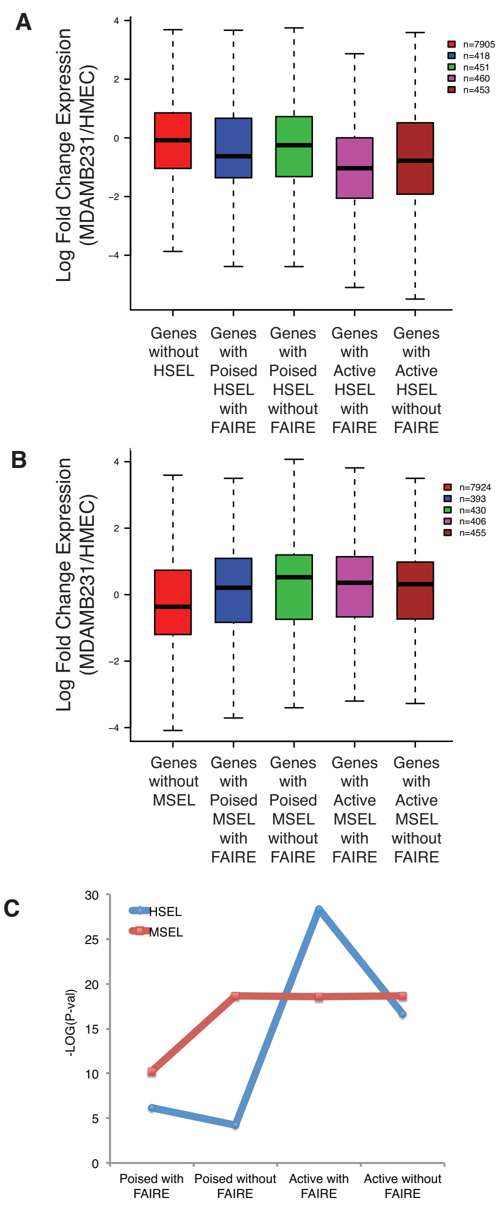
A
Log Fold Change Expression (MDAMB231/HMEC)
4
2
0
-2
-4
n=7905
n=418
n=451
n=460
n=453
Genes without HSEL
Genes with Poised HSEL with FAIRE
Genes with Poised HSEL without FAIRE
Genes with Active HSEL with FAIRE
Genes with Active HSEL without FAIRE
B
Log Fold Change Expression (MDAMB231/HMEC)
4
2
0
-2
-4
n=7924
n=393
n=430
n=406
n=455
Genes without MSEL
Genes with Poised MSEL with FAIRE
Genes with Poised MSEL without FAIRE
Genes with Active MSEL with FAIRE
Genes with Active MSEL without FAIRE
C
-LOG(P-val)
30
25
20
15
10
5
0
HSEL
MSEL
Poised with FAIRE
Poised without FAIRE
Active with FAIRE
Active without FAIRE

Figure S14. Nearby gene expression boxplot for poised and active cell type specific enhancers and FAIRE signal

(A) Log fold change of nearby gene expression boxplot for the poised/active HSEL with and without FAIRE signal (B) Log fold change of nearby gene expression boxplot for the poised /active MSEL with and without FAIRE signal (C) The distribution of significance level for log fold change of nearby gene expression between groups (genes with cell type specific enhancer and genes without cell type specific enhancer). Student t-test was applied between groups in order to calculate p-values: –LOG(p-value) was plotted in the y-axis for significance level.

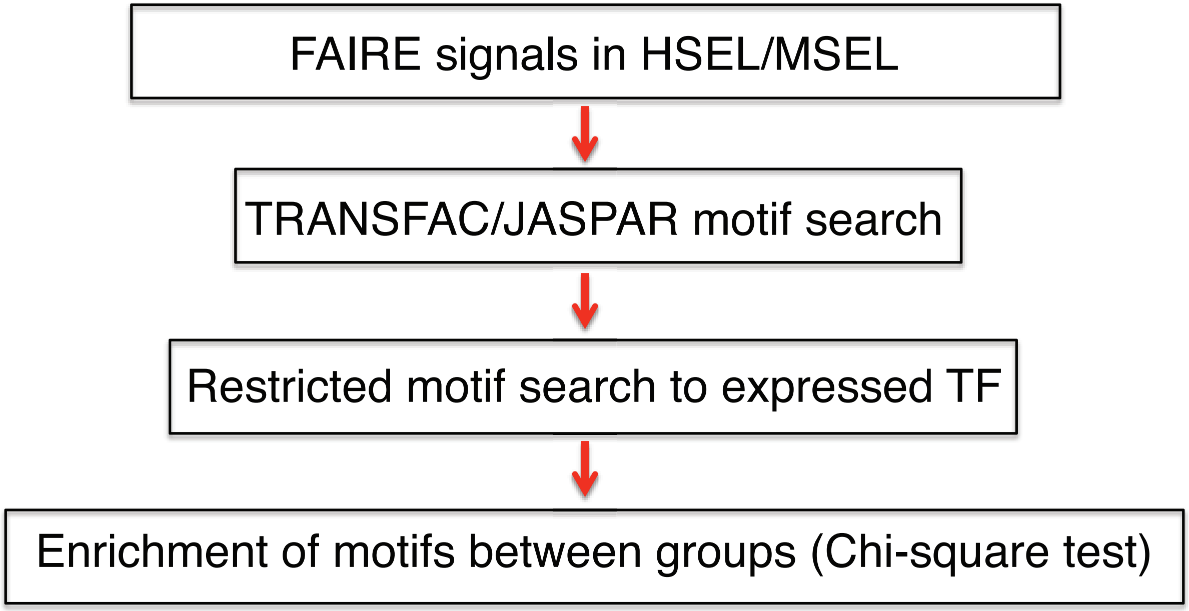

Figure S15. Workflow diagram of transcription factor motif search between enhancer groups

In silico motif search was performed on FAIRE signal in cell type specific enhancers. The motif search was restricted to the expressed transcription factors. The enrichment of motifs between groups was measured by using the chi-square test.

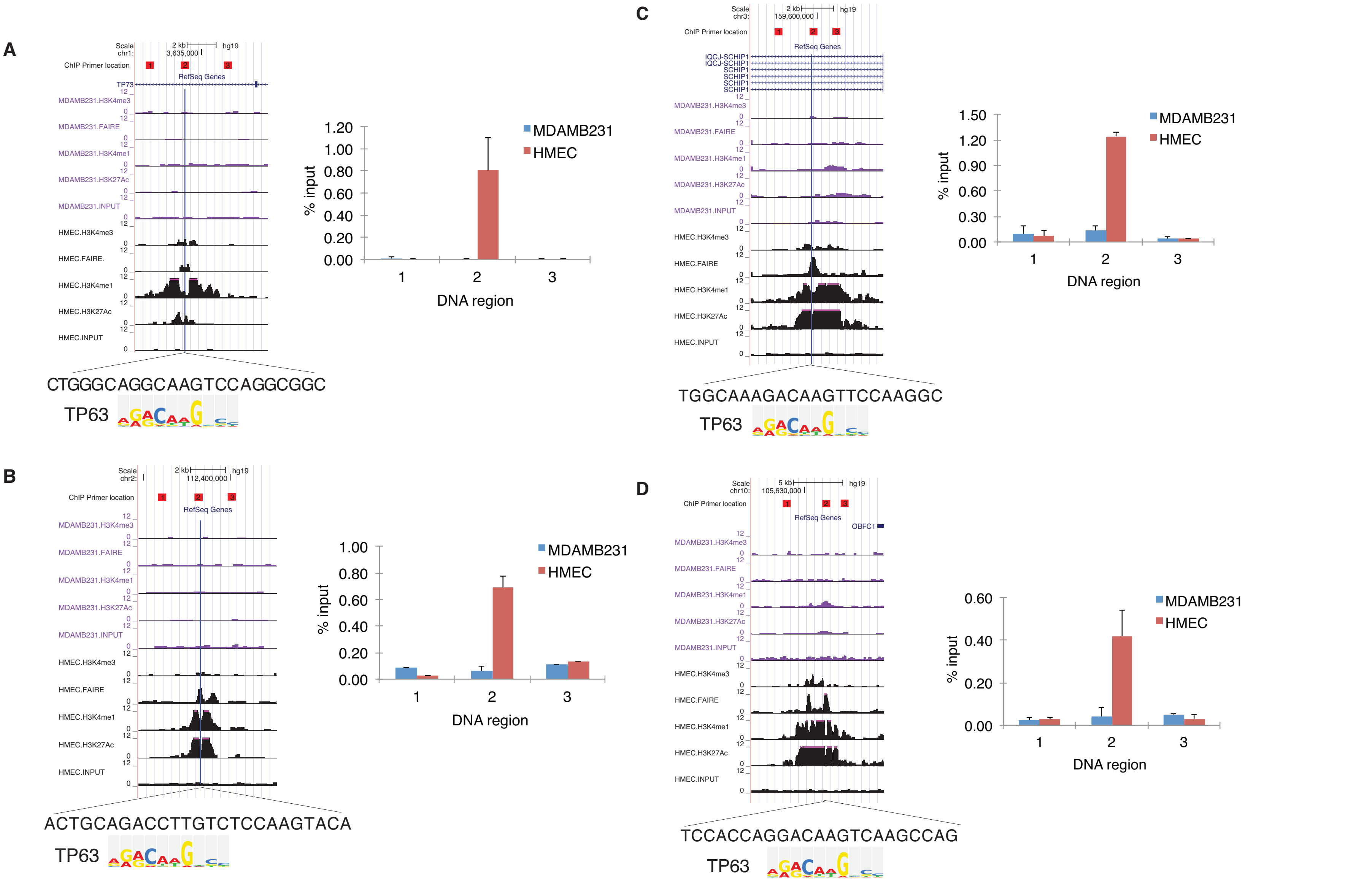
A
Scale
chr1:
2 kb
3,635,000
hg19
ChIP Primer location
1
2
3
RefSeq Genes
TP73
MDAMB231.H3K4me3
MDAMB231.FAIRE
MDAMB231.H3K4me1
MDAMB231.H3K27Ac
MDAMB231.INPUT
HMEC.H3K4me3
HMEC.FAIRE.
HMEC.H3K4me1
HMEC.H3K27Ac
HMEC.INPUT
CTGGGCAGGCAAGTCCAGGCGGC
TP63
% input
1.20
1.00
0.80
0.60
0.40
0.20
0.00
MDAMB231
HMEC
DNA region
B
Scale
chr2:
2 kb
112,400,000
hg19
ChIP Primer location
RefSeq Genes
ACTGCAGACCTTGTCTCCAAGTACA
TP63
MDAMB231
HMEC
DNA region
C
Scale
chr3:
2 kb
159,600,000
hg19
ChIP Primer location
RefSeq Genes
IQCJ-SCHIP1
SCHIP1
TGGCAAAGACAAGTTCCAAGGC
TP63
1.50
1.20
0.90
0.60
0.30
0.00
MDAMB231
HMEC
DNA region
D
Scale
chr10:
5 kb
105,630,000
hg19
ChIP Primer location
RefSeq Genes
OBFC1
TCCACCAGGACAAGTCAAGCCAG
TP63
0.60
0.40
0.20
0.00
MDAMB231
HMEC
DNA region

Figure S16. TP63 binding in breast epithelial cell type specific enhancers.

Examples of the HSEL with TP63 motif, located in the intron of *TP73* gene in 1p36.32 (A), gene desert region in 2q13 (B), intron of *SCHIP1* gene in 3q25.33 (C), and gene desert region in 10q24.33 (D). TP63 ChIP-qPCR experiments at 3 regions (top track of the left genome browser) near each HSEL were performed in MDAMB231 (blue) and HMEC (red) (right). Site-specific ChIPs of TP63 were presented as the average percentage of the input and standard deviations from triplicate by qPCR. Two independent experiments were performed for each case.

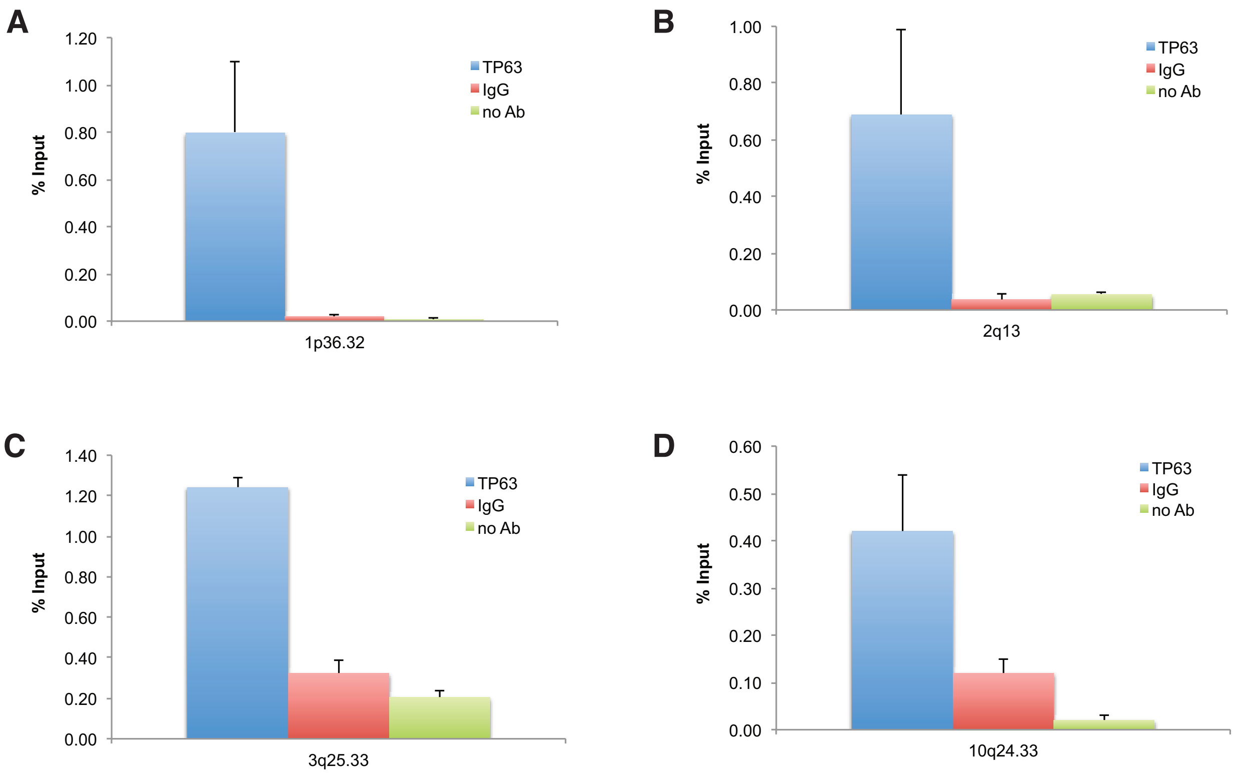

Figure S17. Validation of TP63 binding in breast epithelial cell type specific enhancers in HMEC.

TP63 ChIP-qPCR experiments were performed with two negative controls (IgG antibody and no antibody) at primer 2 regions (see Fig. S16) in examples of HSEL with TP63 motif, located in the intron of *TP73* gene in 1p36.32 (A), gene desert region in 2q13 (B), intron of *SCHIP1* gene in 3q25.33 (C), and gene desert region in 10q24.33 (D).

A

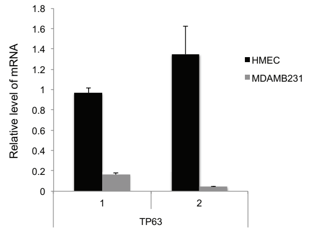

B

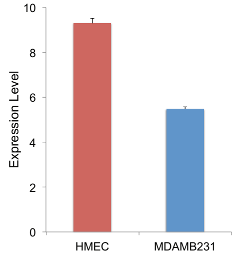

Figure S18. Expression level of *TP63* gene in breast epithelial cells.

Quantitative real-time RT-PCR analysis of *TP63* expression, using two primer sets located in the exon 2 through 5 of *TP63* gene. The expression levels are presented relative to *GAPDH* expression. The error bars indicate the standard deviations from triplicate in HMEC (black) and MDAMB231 (grey) (A). The average expression level of *TP63* genes and standard deviations from triplicate in HMEC (red) and MDAMB231 (blue) cells detected in the affymetrix HG-U133 plus2 microarrays are shown in (B).

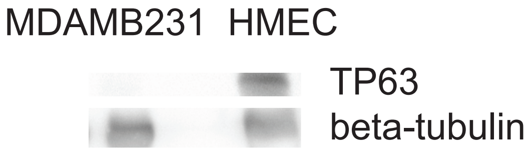

Figure S19. Western blot analysis of TP63

Western blot analysis of TP63 from MDAMB231 and HMEC, with beta-tubulin serving as the loading control.

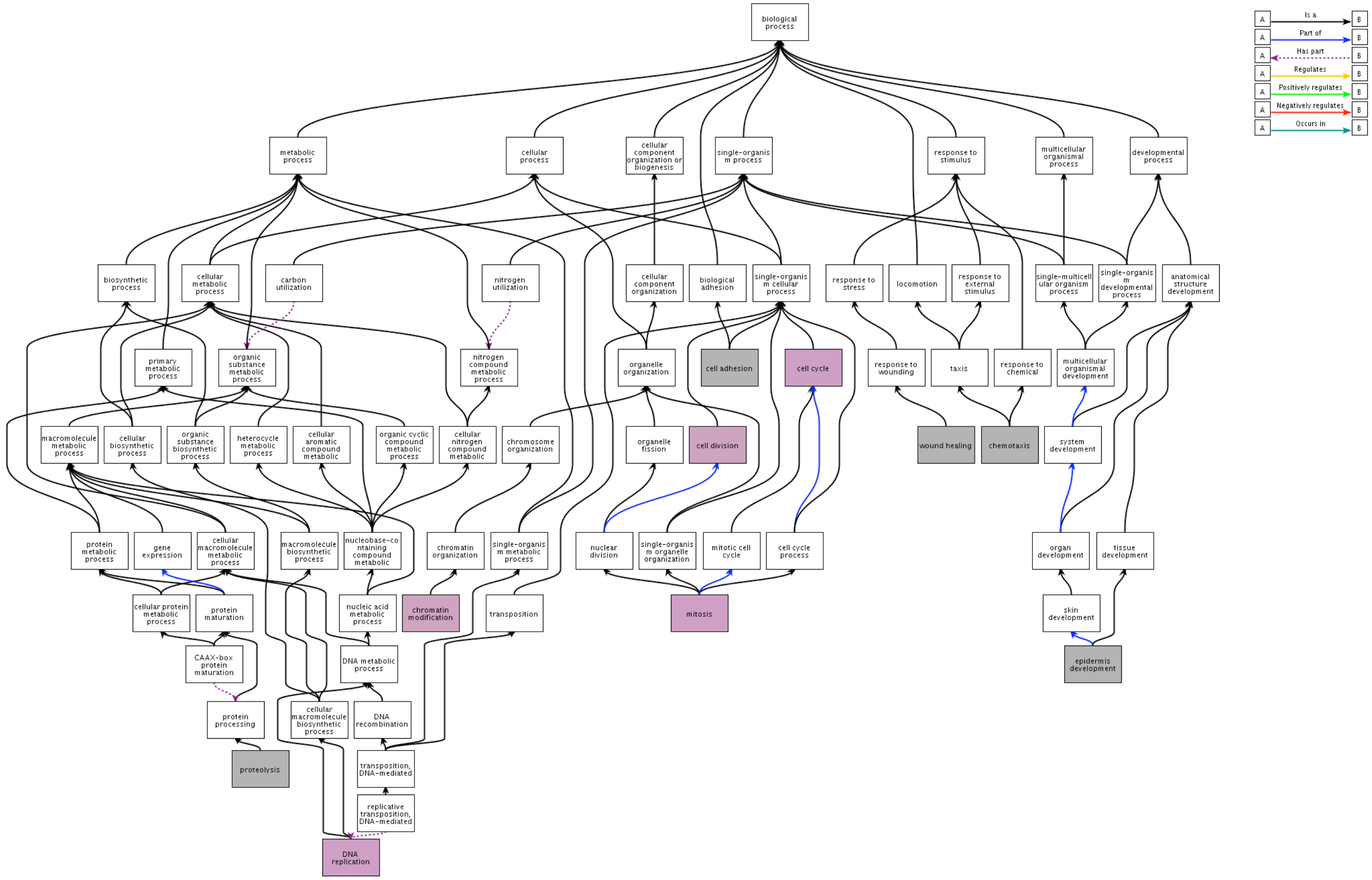

Figure S20. A comparison chart for differentially enriched gene ontology (GO) biological processes

Differentially enriched GO biological processes between the HSEL selected genes and MDAMB231 selected genes are shown. Relationships among GO biological processes are described with different color-coded arrows. GO biological processes that HMEC selected genes belong more are colored in grey box, and purple for the MDAMB231 selected genes.
